# Single particle maximum likelihood reconstruction from superresolution microscopy images

**DOI:** 10.1371/journal.pone.0172943

**Published:** 2017-03-02

**Authors:** Timothée Verdier, Julia Gunzenhauser, Suliana Manley, Martin Castelnovo

**Affiliations:** 1 Univ Lyon, Ens de Lyon, Univ Claude Bernard, CNRS, Laboratoire de Physique, F-69342 Lyon, France; 2 Laboratory for Experimental Biophysics, School of Basic Sciences, Swiss Federal Institute of Technology (EPFL), Lausanne, Switzerland; University of California Irvine, UNITED STATES

## Abstract

Point localization superresolution microscopy enables fluorescently tagged molecules to be imaged beyond the optical diffraction limit, reaching single molecule localization precisions down to a few nanometers. For small objects whose sizes are few times this precision, localization uncertainty prevents the straightforward extraction of a structural model from the reconstructed images. We demonstrate in the present work that this limitation can be overcome at the single particle level, requiring no particle averaging, by using a maximum likelihood reconstruction (MLR) method perfectly suited to the stochastic nature of such superresolution imaging. We validate this method by extracting structural information from both simulated and experimental PALM data of immature virus-like particles of the Human Immunodeficiency Virus (HIV-1). MLR allows us to measure the radii of individual viruses with precision of a few nanometers and confirms the incomplete closure of the viral protein lattice. The quantitative results of our analysis are consistent with previous cryoelectron microscopy characterizations. Our study establishes the framework for a method that can be broadly applied to PALM data to determine the structural parameters for an existing structural model, and is particularly well suited to heterogeneous features due to its single particle implementation.

## Introduction

Many cellular machines, such as the multi-protein structures involved in membrane fission or fusion, transport across membranes, cell division, and more, lie below the resolving power of fluorescence microscopy. Superresolution fluorescence imaging promises to directly reveal their organization *in situ*. Indeed, recent microscopy studies of the midbody [1, 2], centriole [3–6], and nuclear pore [7, 8] have advanced the models for how such machines are assembled.

At the same time, these studies reveal some of the current limitations in interpreting superresolution images. Among the highest demonstrated resolutions are methods based on single molecule localization, variously known as (fluorescence) photoactivated localization microscopy ((f)PALM) or stochastic optical reconstruction microscopy (STORM) [9–11]. These methods use stochastic serial photo-activation of fluorescent dyes within the sample to ensure their sparsity, which enables their precise positioning at the center of each distinct diffraction-limited spot they generate. The position of each emiter is therefore much precised on the final image where each diffraction spot is substituted for by its centroid (or by a smaller spot whose size express the uncertainty of the fit). However, because of the stochastic nature of the method, images are inherently noisy and the amount of information they carry is limited in part by the labeling density, which is limited by the dye targets density inside the sample and because all dyes cannot be read [12]. Furthermore, when the localization precision of single molecules is not much smaller than the size of the object, the structural information content of the image is distorted or obscured by positioning errors. This positioning error is intrinsic to optical imaging and depends mainly on the fluorophore quantum yield. Thus, individual images may often lack sufficient quality to test or build structural models. Instead, significant particle averaging is often necessary to use the data to prove a model [3, 7, 13].

When molecular structures or particles are identical, statistical averaging over a large set of images is a valid way to address these limitations. However, in the most general case, there can be genuine structural variability [14, 15], that may be difficult to interpret when relying on approaches based on population averaging. We propose to address this by introducing a new reconstruction method, which can be applied at the single particle level. This assumes that we have a geometrical parametrization of the structure –an *a priori* structural model–, and that we want to determine its parameters from the PALM images. For instance, the object could be known to be a cylinder whose length and radius we would like to determine: dynamin is an example of a protein that polymerizes into such a structure to induce membrane fission [16]. Our method, a maximum likelihood approach, calculates a score for all possible values of parameters. The score corresponds to the probability of obtaining the observed data from the structure parametrized by this set. Thus, we can identify the highest scoring set of parameters, which corresponds to the most probable structure underlying the measured data.

**Fig 1:**
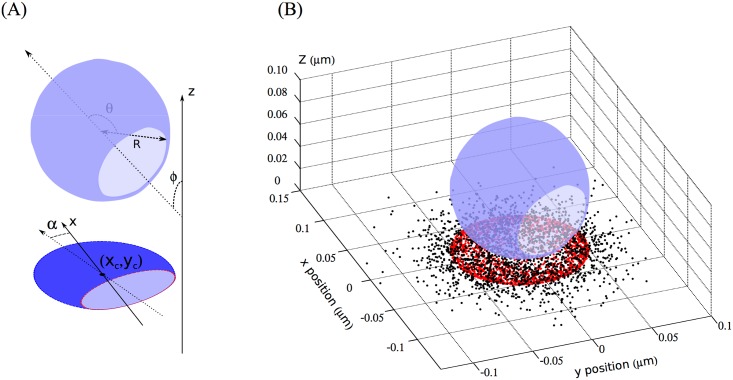
Fitted structure and simulated data. (A) The assumed density of Gag (transparent blue) is shaped as a truncated spherical shell of radius R, completion angle *θ*, and is tilted by an angle *ϕ* with the projection axis (the optical axis). Its projection is distributed around the projected sphere center (*x*_*c*_, *y*_*c*_) and makes an angle *α* with the plan *x*-axis. (B) To simulate palm images, we uniformly sample points in this density, project them on the plane (red dots), and move each of them by a random normal displacement of std. *σ*_*i*_ (black dots) accounting for the superresolution imprecision.

We illustrate the advantages of this approach by validating it on PALM images of budded, immature virus-like particles (VLPs) formed from the fluorescently tagged polyprotein HIV-1 Gag. Cryo-electron microscopy has shown that VLPs are formed from an incomplete spherical protein shell beneath the lipid viral envelope and are highly polydisperse [15, 17]. Gag proteins from individual correctly formed VLPs are therefore expected to lie on spherical shells of variable radii and closure angles (Fig 1), features that would be obscured by particle averaging. We compute the maximum likelihood 3D geometry, thereby estimating the particle radius and protein coverage for individual VLPs. We apply this strategy to both simulated and real PALM data. Comparison between the parameters used as inputs to simulate the data and the output parameters of the reconstruction procedure gives an estimate of the precision reached at a statistical level. This way, we are able to estimate the radius and the closure angle that best explain the measured data from a given particle, and also can extract the uncertainty on this estimation.

The remainder of this article is organized as follows. In the first section, entitled “Theoretical Framework”, we detail the likelihood calculation of a parameter set given a superresolution image and briefly review the maximum likelihood estimator framework. We conclude this section by introducing the geometrical parametrization we subsequently use to model HIV-1 structure. The next section, Material and Methods, details the experimental and numerical methods we used to record, simulate and analyze the HIV-1 PALM images. Results of the analysis of simulated and experimental PALM images are described in the following “Results” section. In the last sections, “Discussion and conclusion”, we analyze the precision and limitation of the maximum likelihood method applied to HIV-1 radii and completions estimation, we compare our results to previously reported cryo-electron microscopy data, and we summarize our findings regarding applications to other objects.

## Theoretical framework

### Typical structure of superresolution data

In (f)PALM or STORM data, each emitter position is determined from its diffraction spot by a centroid-finding algorithm [11, 18, 19]. The uncertainty in position can be calculated theoretically, and is related to the width Σ of the microscope point spread function (PSF) and the number *n*_*i*_ of collected photons from the emitter through $\sigma_{i}∼\frac{\Sigma }{\sqrt{n_{i}}}$ [12] in an ideal microscope. In the most common case of 2D superresolution imaging, the parameter set is then simply (*x*_*i*_, *y*_*i*_, *σ*_*i*_). The more photons a fluorescent probe emits and the higher its signal-to-noise ratio, the more precisely the molecule can be localized and the smaller *σ*_*i*_.

Our aim is to use a probabilistic approach to extract global structural information about a molecular complex from datasets consisting of lists of molecular positions and uncertainties, (*x*_*i*_, *y*_*i*_, *σ*_*i*_).

### Likelihood calculation

Consider the spatial distribution of emitters. We assume that an *a priori* 3D-density *d*_0_ models this distribution. We will hereafter refer to this model as “the structure”. The structure is defined by a set of parameters that are denoted $\vec{\beta }$ (in the case of VLPs, these parameters include radius, completion angle, and spatial orientation). The first step of the likelihood calculation is to evaluate the probability of measuring an emitter at (*x*_*i*_, *y*_*i*_) with uncertainty in position *σ*_*i*_, given the spatial density of emitters $d_{0}\left[\vec{\beta }\right]$. The probability of measuring an emitter at a given position is $d_{p}\left[\vec{\beta },\sigma_{i}\right]\left(x_{i},y_{i}\right)$, the 2D projected density of emitters at this location blurred by the position uncertainty. To evaluate this “blurring” we need to determine the probability for an emitter to be located at a given distance from its localization position. We call this probability density the blurring function (bf). By definition the blurring function has the position uncertainty *σ*_*i*_ as its characteristic length. Following Mukamel et al. [12], the 3D density prior projection is obtained by convolving the density of emitters *d*_0_ with the blurring function:

$$d_{\text{bf}}\left[\vec{\beta },\sigma_{i}\right]\left(\vec{r}\right)={∭}_{-\infty }^{+\infty }d_{0}\left[\vec{\beta }\right]\left(\vec{r_{1}}\right).\text{bf}\left[\sigma_{i}\right]\left(\vec{r_{1}}-\vec{r}\right).\text{d}\vec{r_{1}}$$ (1)

For simplicity, we take a Gaussian of width *σ*_*i*_ to be the blurring function. We have chosen the *z*-axis to be the optical axis of the microscope. As a consequence, the projected density at (*x*_*i*_, *y*_*i*_) is given by:

$$d_{p}\left[\vec{\beta },\sigma_{i}\right]\left(x_{i},y_{i}\right)=\int_{-\infty }^{+\infty }d_{\text{bf}}\left[\vec{\beta }\right]\left(x_{i},\;y_{i},\;z\right).\text{d}z$$ (2)

By using a modified experimental setup [20–23] the *z*_*i*_ position of single emitters can be extracted –although typically with greater uncertainty $\sigma_{i}^{z}$. The aforementioned 2D procedure extends to the 3D case by simply ignoring the projection. The complete dataset is then: (*x_i_*, *y_i_*, *σ_i_*, *z_i_*, $\sigma_{i}^{z})$. The 2D calculations above still apply but with an anisotropic blurring function and no projection afterwards. The 3D localization probability density is found by convolving the structure with a gaussian of width *σ*_*i*_ in the lateral directions and $\sigma_{i}^{z}$ in the axial one. This way, we directly obtain the 3D blurred density of emitters [24]: $d_{\text{bf}}\left[\vec{\beta },\sigma_{i},\sigma_{i}^{z}\right]\left(x_{i},y_{i},z_{i}\right)$. All that follows can be applied to this 3D localization case by substituting $d_{\text{bf}}\left[\vec{\beta },\sigma_{i},\sigma_{i}^{z}\right]\left(x_{i},y_{i},z_{i}\right)$ for $d_{p}\left[\vec{\beta },\sigma_{i}\right]\left(x_{i},y_{i}\right)$ and will not be further emphasized for the sake of readability. Note that in the projection scheme Eq 2, it was implicitely assumed that the error in location of the emitter are identical beyond the focal plane. While the average position of these emitters would remain the same in the 2D projection, the error on their position estimate will be larger as emitters move beyond the focal plane due to the spread of their photons along larger spots. This effect is neglected in the present approach for the sake of tractability.

From another perspective, given a measurement (*x*_*i*_, *y*_*i*_, *σ*_*i*_), the quantity $d_{p}\left[\vec{\beta },\sigma_{i}\right]\left(x_{i},y_{i}\right)$, is also the likelihood that the distribution of emitters is defined by $\vec{\beta }$. Indeed, this is the conditional probability density of localizing an emitter at position (*x*_*i*_, *y*_*i*_) given a structure defined by $\vec{\beta }$ and an uncertainty in measured position *σ*_*i*_.

Since a dataset consists of ensembles of molecular positions, we need to extend the previous calculation to obtain the joint probability for the entire set of localizations. Individual localization events are assumed independent of each other. The likelihood for the whole set of measurements to be defined by $\vec{\beta }$ can therefore be written as the product of likelihoods for a single measurement:

$$L\left(\left\{x_{j},y_{j},\sigma_{j}\right\}\right)=\prod_{j}d_{p}\left[\vec{\beta },\sigma_{j}\right]\left(x_{j},y_{j}\right)$$ (3)

This result is often presented in its logarithmic form, the log-likelihood or score function:

$$\text{logL}\left[\vec{\beta }\right]\left(\left\{x_{j},y_{j},\sigma_{j}\right\}\right)=\sum_{j}\log\left(d_{p}\left[\vec{\beta },\sigma_{j}\right]\left(x_{j},y_{j}\right)\right)$$ (4)

The optimal choice of geometric parameters ${\vec{\beta }}_{*}$ to describe the observed data is the set which maximizes the likelihood function *L* or equivalently the log-likelihood [25]. In other words, ${\vec{\beta }}_{*}$ defines the distribution of emitters that maximizes the probability to have such a measurement. This condition is written as:

$$\vec{\beta_{*}}=\underset{\vec{\beta }}{\text{argmax}}\left(\text{logL}\left[\vec{\beta }\right]\left(\left\{x_{j},y_{j},\sigma_{j}\right\}\right)\right)$$ (5)

As proposed in [26], the observed Fisher information matrix J can be used to evaluate an upper bound on the precision of the parameter determination. For the sake of simplicity, we use this estimation as the error bars for the fit. Under general regularity conditions, the observed Fisher information matrix J is the negative of the log-likelihood Hessian matrix at its maximum which has indeed the dimensions of the inverse of a covariance matrix. In other words, the confidence interval is given by the curvature of the log-likelihood function around its maxima:

$$H_{ij}={\left.\frac{\partial^{2}\text{logL}\left[\vec{\beta }\right]}{\partial \beta_{i}\partial \beta_{j}}\right|}_{\vec{\beta *}}\;\text{and}\;J=-H\approx {\text{Cov}}^{-1}\left(\vec{\beta }\right)$$ (6)

Remarkably, the blurring function and the *a priori* geometrical model for the spatial localization of emitters are the only necessary inputs to conduct the reconstruction procedure outlined in this section.

### Analysis of immature HIV-1 VLP imaged by PALM

HIV-1 VLPs are good candidates for our reconstruction approach at the single particle level, because of the high size polydispersity reported in the literature [15]. In the case of immature HIV-1 VLPs, Gag proteins are expected to form an incomplete protein lattice. In this case, we choose a simple *a priori* structural model showed in Fig 1: a truncated sphere of radius R and protein coverage angle *θ* (designated below as the “completion angle”). This structure depends on seven parameters: one for the radius of the sphere, three for its center position, two for its orientation (Euler angles to orient the symmetry axis of the structure), and one for its completion. Once projected into 2D, we reduce the degrees of freedom by one (the axial center coordinate). We parametrize the structure by *β* = (R, *θ*, *ϕ*, *α*, *x*_*c*_, *y*_*c*_), respectively radius, completion angle, tilt angle between the microscope optical axis and the model symmetry axis, rotation angle in focal plane, and projected center coordinates.

We can further reduce the complexity of this problem by taking advantage of the axisymmetry of the model, and of its quantities that are quasi-invariant under the gaussian convolution (therefore hardly affected by the positioning error introduced by the Gaussian blurring function). As proposed in [27], the first geometrical moments of the emitter distribution define the center position and one orientational angle, and therefore allow us to center the points distribution on its center of mass ${\vec{X}}_{\text{CM}}$ and reorient it in the focal plane according to its principal axis. Without loss of generality centering and orientation are calculated analytically (see ${\vec{X}}_{\text{CM}}$ and *α* calculation in supplementary information S1 File).

Using this reduced coordinate system, we compute the optimal ${\vec{\beta }}_{*}$ and its uncertainty by finding the maximum log-likelihood and its local curvature as given by Eqs 5 and 6. This step is performed numerically using a quasi-Newton algorithm which is computationally expensive. To speed up the computation, we use an average value $\bar{\sigma }=\sqrt{\sum_{j}\sigma_{j}^{2}/N}$, where *N* is the total number of emitters, instead of each emitter localization uncertainty *σ*_*j*_:

$$d_{p}\left[\vec{\beta },\sigma_{j}\right]\left(x,y\right)\Rightarrow d_{p}\left[\vec{\beta },\bar{\sigma }\right]\left(x,y\right)$$ (7)

This approximation allows us to calculate a single convolution for all of the localized fluorophores instead of evaluating the convolution values *N* times, once for each of the localized fluorophore positions, which considerably speeds up the computational time (see supplementary information S1 File). As a drawback, the broader the distribution of uncertainties {*σ*_*j*_}, the less appropriate our approximation.

For several data sets, either simulated and experimental, two local maxima compete in the parameter range (*θ*, *ϕ*) ∈ [0, *π*] rad ×[0, *π*/2] rad. One is found for a small completion angle *θ* ∈ [0, *π*/2] rad, a small viewing angle *ϕ* ≈ 0 and a high radius value whereas the other is found for *θ* ∈ [*π*/2, *π*] with no restriction on *ϕ* and at a smaller radius value. Computations of the log-likelihood values over the full domain on simulated data showed that this situation arises when the input tilt orientation *ϕ* is small, otherwise no secondary extremum is seen. Since we work on VLPs that have fully budded, we further assume more than half completion of the Gag shell and therefore we restricted our parameter space to a range *θ* ∈ [*π*/2, *π*] rad where only one maximum is found.

## Materials and methods

### Cell culture, transfection and VLPs extraction

African green monkey kidney cells (Cos7) were cultured in DMEM supplemented with 10% FBS (Sigma Aldrich). For VPL production 600,000 cells were grown in T75 flasks and transfected with 34 *μ*g of Gag-mEos2 plasmid (described in [14]) and 100 *μ*l FuGene6 (Roche Diagnostics) in a total volume of 1 ml DMEM without FBS incubated for 15 min. 48 hours post transfection the supernatant was collected from the cells and filtered through 0.45 *μ*m filters. For VLP extraction the supernatant was centrifuged over a 20% sucrose gradient at 27000 rpm for 2 hours at 4°C. The pellet was dissolved in filtered PBS and the VLP solution was directly used for imaging or stored for not more than 24 hours at 4°C prior to imaging. For imaging, poly-L-lysine coated coverslips containing 100 nm Au fiducial markers were incubated with VLPs for 1 hour at 4°C, rinsed with PBS and directly used.

### Superresolution imaging

VLPs were imaged using a Zeiss Axio Observer D1 inverted microscope, equipped with a 100×, 1.49 NA objective (Zeiss). Activation and excitation lasers with wavelengths 405 nm (Coherent cube) and 561 nm (Crystal laser) illuminated the sample in total internal fluorescence (TIRF) mode. We used a four color dichroic 89100bs (Chroma), fluorescence emission was filtered with an emission filter ET605/70 (Chroma) and detected with an electron-multiplying CCD camera (iXon+, Andor Technology) with a resulting pixel size of 160 nm. For each region of interest, typically 30000 to 40000 images of a 20.5 x 20.5 *μm*^2^ area were collected with an exposure time of 30 ms. The irreversible photoactivatable protein mEos2 was activated with low continuous 405 nm laser intensity to guarantee very sparse activation and minimize blinking, and excited with 561 nm laser intensity of ≈ 1 kW.cm^−2^. Molecules were localized using Peakselector (IDL, courtesy of Harald Hess). The single molecule localization procedure consisted of the following steps: a) fluorescent intensity peaks were detected on each image, b) each peak was fitted to a two-dimensional Gaussian by nonlinear least-square fitting to obtain x and y coordinates as well as the localization precision, c) images were dedrifted using Au fiducial markers embedded into the cover glass (Hetzig.com) (see S11 Fig for details), d) localizations detected within less than the measured mean localization precision (typically between 17 and 24 nm) in space and 300 ms in time were grouped to account for blinking of mEos2. For mEos2, we find on average 3.5 switching cycles which is insufficient for a precise determination of the localization precision (S12 Fig). Hence in the present study, we rely on the theoretical localization precision [28]. One grouped molecular position is counted as one Gag-mEos2 protein [14].

### Superresolution analysis

The analysis presented in the Material and Methods section is focused on viral particles that fully escaped the cellular membrane. The data consisted of 33 sets of *N* superresolved position triplets {*x*_*j*_, *y*_*j*_, *σ*_*j*_}, *N* ranging from 714 to 3302 proteins and $\bar{\sigma }$ ranging from 15 nm to 21 nm. The distribution of radii estimated through the MLR procedure described above was created summing normalized Gaussian distributions centered on the estimated value, with variance given by the first diagonal coefficient of the corresponding observed Fisher information matrix (Eq 6). Hence a radius estimated with a large error bar contributes on a large interval and each particle has the same weight in the total. The resulting distribution shows a strong peak at R = 45 nm and an extended tail for larger radii. One object in the sample exhibits a radius much larger than the mode (up to twice the main peak radius). The outlier particle, shown in supplementary S5 Fig, exhibits an elongated shape and is likely to be an aggregate or an ill-formed particle, and is thus excluded from the analysis. The distribution of estimated completion angle is built following the same procedure, but as the angular interval is finite, the normalization was made on [*π*/2, *π*].

### Simulation of super resolution data

Simulated superresolution data were produced with MATLAB^®^ by randomly sampling simulated label positions $\left(x_{i}^{0},y_{i}^{0},z_{i}^{0}\right)$ for different 3D-density-priors for the tagged-Gag layer shape (truncated spherical shell as shown in [15]) or control reference states (fully complete spherical shells). An imprecision for each position *σ*_*i*_ was then chosen following a Gaussian shape distribution that mimics the experimental PALM-data localization uncertainty distribution (*μ* = 20 nm, std = 5 nm). The superresolution fitted position is deduced from the sampled position $\left(x_{i}^{0},y_{i}^{0}\right)$ by adding a random displacement in the plane (*δx*_*i*_, *δy*_*i*_) with normal isotropic distribution $\delta_{i}∼N\left(0,\sigma_{i}\right):\left(x_{i},y_{i}\right)=\left(x_{i}^{0}+\delta x_{i},y_{i}^{0}+\delta y_{i}\right)$ (see Fig 1). The exponential decrease of the exciting intensity in TIRF microscopy and subsequent diminution of positioning precision with depth can be neglected since the TIRF depth is at least of the size of the VLPs (≃100 nm): the effect is thus at most comparable to the stochastic variation of photon yield of the fluorophores before they bleach, and positioning precision can be assumed homogeneous at all the positions. Furthermore, this effect being isotropic in the optical plan, it does not affect the symmetry of the image in 2D that is crucial for our centering and orientation methods.

We computed Eq 2 numerically. This step is the most computationally intensive, so we used Fast Fourier Transform (FFT) to transform the Gaussian convolution into a simple product and speed up the calculation (see S1 File). Once we were able to evaluate the likelihood function for any parameter set, the optimization problem was numerically solved using the “interior-point” algorithm of MATLAB^®^ “fmincon” solver. The solver was started with different initial values to check the robustness of its convergence to a maximum. The Hessian matrix at maxima positions was deduce from the fit of a quadratic form using 1000 evaluation points in the neighborhood of the maximum to assure robustness towards numerical noise at low scale (rounding errors).

## Results

### Reconstruction from simulated PALM images

To evaluate the performance of our maximum likelihood reconstruction (MLR) procedure, we simulated PALM images of truncated spheres of known geometrical parameters (R, *θ*, *ϕ*). We thus provided as an input 300 simulated datasets composed of 1500 points each with an uncertainty in position of 20 nm, comparable to the experimental datasets (Fig 1). We could therefore compare the computed optimal structure to the “ground truth” input structure and estimate the precision of our method from these differences. This also provides a test for the proposed confidence estimation calculated from the observed Fisher information matrix. The first two parameters are associated with the geometry of the virus-like-particles (radius R and completion of the sphere *θ*), while the last one is an orientation parameter (tilt angle *ϕ* of the particle with respect to the optical axis). This last degree of freedom, although necessary to fit the model properly, contains no physical or biological information and will not be discussed. The parameters of the simulated data were randomly drawn with a uniform probability in the interval: (R, *θ*, *ϕ*) ∈ [30, 80] nm ×[*π*/2, *π*] rad ×[0, *π*/2] rad.

**Fig 2:**
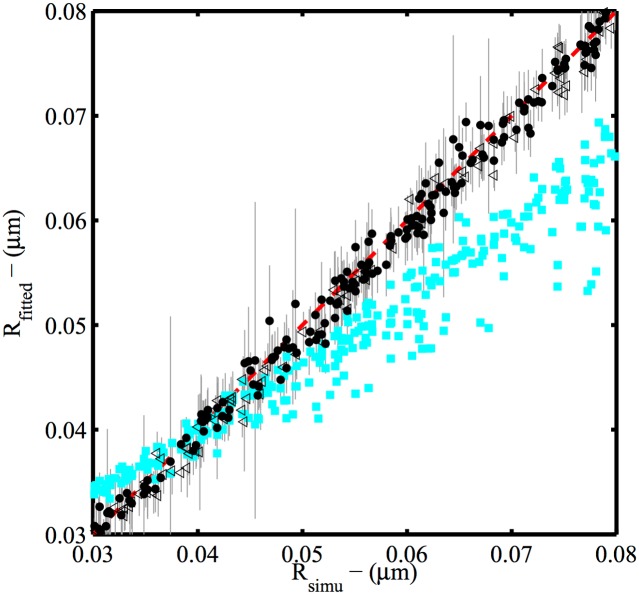
Maximum Likelihood Reconstruction efficiency on radii. The maximum likelihood method is compared to a simpler method based on the mean distance from the center of mass. The MLR-estimates of radii from 300 simulated superresolution data-sets R_*_ (black dots and triangles) plotted as a function of the ground truth values R_0_ follow on average the perfect estimator behavior *R*_simu_ = *R*_fitted_ (dashed red line). Particles for which the planar orientation *α* is ill-determined (quasi-isotropic distribution with Δ*α* > *π*/10 -see Eq S9 in supplementary information S1 File) are shown as empty triangles. Computation of the observed Fisher information matrix gives an estimate of the precision reach by the estimator for each VLP (grey error bars). For the sake of comparison, a basic statistical estimator using no specific structural information, the mean distance to the center of mass estimator $R_{1}=<\left\|{\vec{r}}_{i}-{\vec{r}}_{\text{CM}}\right\|>$, is also shown (cyan squares).

The comparison between actual and estimated particle radii including the estimated error bars are shown in Fig 2. We found that the particle radius estimation *R*_fitted_ is in good agreement with the ground truth value. The standard deviation of the difference Δ*R* = *R*_fitted_ − *R*_simu_ was *σ*_Δ*R*_ = 1.3 nm while the average error, or bias $\bar{\Delta R}$ was < 1 nm). Thus 95% of the simulated particles have been reconstructed with an error on the radius smaller than 3 nm. Also, this error is independent of the true radius, unlike in other common simple procedures based on averages over the distribution of emitter positions. Such geometry-dependent error clearly affects the basic 2D radius estimator –the mean distance to the center of mass $R_{CM}=<\sqrt{(}{\left(x_{j}-<x_{j}>\right)}^{2}+{\left(y_{j}-<y_{j}>\right)}^{2})>$–shown for comparison. This simple estimator is biased and highly variable because completion and tilt degrees of freedom are not taken into account. Furthermore it is not able to distinguish anything smaller than or of the order of the positioning precision as does our procedure that takes this information into account. Remarkably, this result shows that the radius of the particle can be deduced at the single object level from our reconstruction procedure with an accuracy better than the microscope precision ($\bar{\sigma }\approx 20$ nm) by one order of magnitude in the conditions of our simulation.

The variance on each fitted parameter is estimated from the diagonal element of the observed Fisher information matrix given in Eq 6. The average variance is equivalent to an estimated uncertainty (standard deviation) of 3.2 nm and constitutes an upper bound of the measured estimation error in good agreement with its order of magnitude. The details of the error distribution are given in S4 Fig. At the level of individual objects the same observation holds: error bars estimated from the observed Fisher matrix are an upper bound of the effective error.

**Fig 3:**
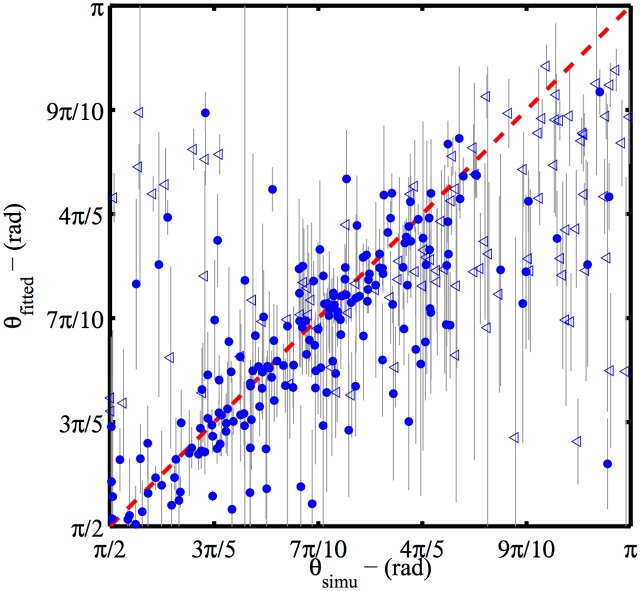
Maximum Likelihood Reconstruction efficiency on completion. Estimated completion values (zenithal angles) of 300 simulated superresolution data-sets determined by the MLR method are plotted against ground truth values (symbols). Particles for which the planar orientation angle *α* is ill-determined (quasi-isotropic distribution with Δ*α* > *π*/10 -see Eq S9 in supplementary information S1 File) are shown as empty triangles. An ideal estimator would give *θ*_measured_ = *θ*_real_ (red dashed line). Calculations of the Fisher information matrix are used as estimates of the precision reached by the estimator for each VLP (grey error bars). For the figure to be readable, plotted error bar are only a third of the estimated std.

We performed a similar analysis to quantify differences between the actual and estimated values for the Gag shell completion from the MLR on the same simulated data (Fig 3). We observed a standard deviation *σ*_Δ*θ*_ = *π*/10 rad. However, In contrast to the MLR values for the radius, the standard deviation is no longer independent of the completion of the Gag shell. The best agreement between simulated completions and MLR results is obtained for *θ* between 2*π*/3 rad and 5*π*/6 rad. Values outside this range show both a larger systematic average bias and larger standard deviation. Part of this observation is explained by the partial degeneracy between the two boundaries: the density variations of a complete sphere (*θ* = *π* rad) or a half sphere (*θ* = *π*/2 rad) viewed from the top (*ϕ* ≃ 0 rad) are indistinguishable. Furthermore large completion results in low asymmetry of the position distribution. Combined with positioning error and small sampling, the symmetry information might be insufficient to properly orient the point distribution in the plane and the completion estimated on a poorly oriented object might be ill-determined. This second interpretation argues for a careful treatment of the completion results of data sets with unclear symmetry, for which only the fitted radius is meaningful.

Variances calculated from the observed Fisher information matrix on the completion angle are very often much larger that the effective errors *θ*_simu_ − *θ*_fitted_ and spread over the whole value interval. Again, the variance estimated from the Fisher Information matrix appears as an upper bound in the order of magnitude of the error, but it is much less useful since the size of the search interval is in the same range. The average variance is equivalent to an estimated uncertainty (standard deviation) of 0.57 rad whereas the effective error is 0.24 rad. The details of the error distribution is given in S4 Fig.

### Reconstruction from PALM images of budded HIV-1 VLP

**Fig 4:**
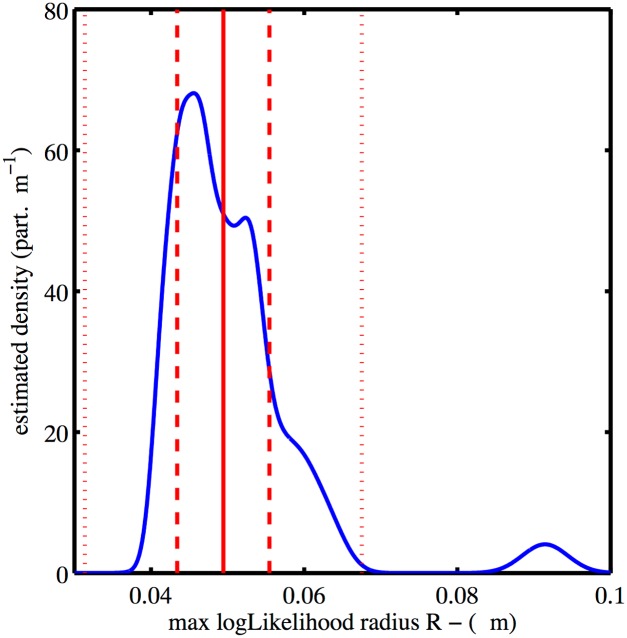
Radii distribution of the truncated spheres-MLR estimated from the experimental PALM-data consisting in *n* = 33 VLPs. The mean value as well as intervals of ±1 and ±3 the standard deviation are shown (red plain line and red dashed lines respectively). Each VLP contribution is a normalized gaussian centered on the estimated radius and whose variance is given by the inverse of the observed Fisher matrix. The second peak *R* ≃ 90 nm is due to a particle that is likely an aggregate (see S5 Fig) and is not taken into account in the mean and variance.

**Fig 5:**
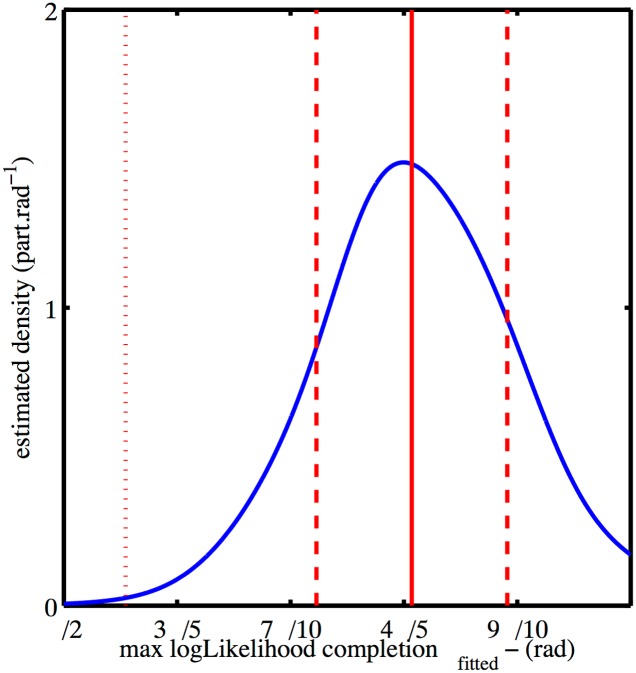
Distribution of estimated completion angles given by the MLR method on the experimental PALM-data (*n* = 33 VLPs—blue), mean value and interval of ±1 and ±3 the standard deviation are shown (red plain line and red dashed lines respectively). Each VLP contribution is a gaussian centered on the estimated radius and whose variance is given by the inverse of the observed Fisher matrix. As acceptable completion angle lay in [*π*/2, *π*], only this part of the gaussian is considered and normalized.

The MLR procedure was applied to analyze PALM images of purified immature HIV-1 Virus-Like Particles (VLPs) produced by Cos 7 cells (see Materials and Methods), applying the same truncated sphere model used for the simulated data. The distribution of estimated radii, Fig 4, had a major peak with mean and standard deviation of 49 nm and 6 nm respectively and a second isolated peak due to a single cluster. The distribution of completion angles estimated from the data is shown in Fig 5. This distribution shows a peak located at *θ* ≈ 4*π*/5 rad, with a standard deviation of *π*/10 rad.

## Discussion and conclusion

We demonstrate in the present work that images from microscopies such as (f)PALM and STORM, when combined with a maximum likelihood-based reconstruction procedure can extract structural information with nanometer precision at the single object level. We highlight that this technique allows us to estimate the most probable structural parameters for every object imaged. This is particularly useful when the complexes to be imaged are heterogeneous.

In this work the modeling of the imaging process was simplified and translated in an effective procedure that would describe a theoretically perfect microscope. Discrepancies between the simulated precision and the experimental situation are at risk whenever factors of error are not taken into account in the simulations. Concerning the physics of imaging the following optical aspect is worth mentioned: in the TIRF configuration used to image the particle in many cases including ours, the excitation field decreases with depth. As a consequence, the photon yield and the positioning precision also decreases with depth. We did not take this effect into account on the simulated images. We basically took into account precision fluctuations by randomly assigning the measurement precision from typical distribution to each sampled position. The estimation method depicted in this work is typically designed for objects whose size is of the order of the measure uncertainty up to a few times this value and so smaller or at worse comparable to the penetration depth of the TIRF field (estimated at several hundreds of nanometers compared to HIV typical diameter of 140 nm). As mentioned in the Materials and Methods section, the modulation of the emission intensity by the excitation intensity decay should be typically dominated by the relative fluctuations the stochastic fluctuations of individual fluorophore photon yield that we simulated. In the case of using the simulation method on larger objects, the positioning precision would certainly convey information about the label depth and the shape of the image might also be altered to a larger extend. In this case, to obtain the precision of likelihood estimation procedure by simulations would require to generate more realistic images.

We applied MLR to a specific test case: immature HIV-1 VLPs imaged using PALM. The fluorescently tagged viral proteins Gag form an incomplete shell enclosed by a lipid membrane, as observed by several groups using cryo-EM [15, 29]. Thus, we chose as a model a truncated sphere of radius R and completion angle *θ*. The reconstruction procedure was first tested using simulated PALM images of such truncated spheres with known input parameters. Remarkably, we found that the MLR approach is able to estimate the radius of particles with an excellent accuracy (≈ 1.3 nm), much smaller than the localization uncertainty of a single emitter (≈ 20 nm). In particular, the MLR estimation for the radius gives a better result than standard radial estimators such as the mean radius. This observation makes sense, since in MLR, the data are analyzed given the information of a constrained geometry set by the model.

Conversely, we found that the estimation of sphere completion using the reconstruction procedure is less successful. We interpret this observation in the following way: the projection associated with a tilt close to *ϕ* ≃ 0 generates a degeneracy between mid- and full closure. It also renders the determination of the orientation of the particle in the focal plane more difficult, which negatively impacts the validity of the completion estimation. Those effects account for the large spread at the extremes of the interval. Perhaps some of the particles belonging to each ends of the interval are falsely identified as belonging to other part of the interval. This leads to an increase at the center of the interval which results in an overestimation of the number of particle with completion in [2*π*/3, 5*π*/6] and an underestimation of the number of particles close to full completion.

Interestingly, the aforementioned reduced efficiency for the estimation of completion at the single object level only partially distorts an ensemble measurement. Indeed, we observed with the simulated data that for completion values uniformly sampled on an interval [*θ*_−_, *θ*_+_], the MLR estimation provides completion values centered on this interval, and marginally spread values outside of this range. This information can be therefore used to define roughly the range of variation for the completion of the imaged objects (see S3 Fig). The measurement of 3D data could also be used to reduce the degeneracy.

Based on the previous results concerning the rather uncertain determination of orientation and completion, it might be tempting to consider that these parameters are irrelevant. In order to address this question, we simulated a new set of incomplete particles, and we analyzed these data with a complete sphere model (obtained with the truncated sphere model by imposing a completion of *θ* = *π*). The results of radius reconstruction in this case is shown in S6 Fig. It is observed that the error on the radius determination is strongly dependent on the value of initial completion and orientation. Not surprisingly, the smallest errors are obtained for almost full completion *θ* ∼ *π*. However the error can be as high as 5–10 *nm* for certain values of *θ* − *ϕ*, and the bias is therefore much larger than in the reconstruction procedure using the truncated sphere model ($\bar{\Delta R}<1$ nm) and the dispersion is also large (≃2.9 nm compared to 1.3 nm). This shows that although the completion and orientation are determined in a poorer way than the radius, these parameters are still essential in order to get a more accurate determination of the radius when the actual structure is likely to be incomplete.

We also analyzed PALM images obtained on immature HIV-1 VLP using MLR in the light of our simulations. The distribution of observed radii (Fig 4) is characterized by a mean and standard deviation of 49 nm and 6 nm respectively. The fluorescent protein tags that are imaged are attached to the Gag N-terminus, located at the inner surface of the Gag layer. Literature values from cryo-EM measurements give VLP sizes in terms of outer diameters. To take this into account for the purposes of comparison, we add the reported Gag length of 25 nm in immature VLPs [30] to the size of the measured particles. The adjusted mean radius of HIV-1 VLPs in our measurement is on average 74 ± 6 nm, consistent with values from cryo-EM of 66 ± 9 [31], and 65 ± 17 nm [15]. Moreover, our analysis was able to identify an ill-formed HIV-1 VLP among the others (see S5 Fig) by its abnormal effective radius (the formation of morphologically aberrant VLPs from Gag proteins fused to fluorescent proteins has been reported [32]).

We were also able to estimate the completion distribution. The main peak of the completion distribution is found at approximately *θ* = 5*π*/6 rad. As discussed previously, the value of the peak is only indicative of a range of completion around this value for the imaged VLPs. We can conclude, however, that the shape of the distribution obtained from real data is not qualitatively consistent with MLR completion estimations for complete spheres, or for a uniform completion distribution on the interval (see S3 Fig). Our finding of incomplete closure is consistent with the 2/3 surface coverage reported in [15].

The MLR method principle proposed in this work can be broadly applied to analyze at the single object level other features imaged by SR-microscopy with known parameterizable shape. Notice that the likelihood value is meant to choose the best parameter set within a model and not to allow the comparison between two different models designed in order to describe a common set of data as one could naively try. Other methods, which are still debated, have been proposed instead for this purpose like the use of Akaike criterion. More informations can be found in references [33].

Additionally, it is not possible to extract from the data more information than it carries, even as the optimization procedure always provides a result. There is then two criteria to judge the relevance of this result: first the statistical agreement between the estimated structure and the measured positions (the “goodness of fit”) and second the amplitude of fluctuations in the estimated parameters allowed by the measure stochasticity. In this paper we restricted ourself to situations where the analysis is satisfying by simply assuming that the model reasonably describe the situation of the measure. However we propose in supplementary information (S1 File) a test example of the goodness of the fit obtained by the present method using the approach of Kolmogorov-Smirnov statistical test of distribution comparison adapted to multidimensional case [34]. We applied such a method to our maximum likelihood reconstruction of complete spheres, (see supplementary information S1 File) in order to show its typical implementation and results to the interested reader. The method is indeed able to point out the distributions of localizations that differ significantly from the adjusted structure under the hypothesis of a random sampling. We however detailed different test to judge of the estimation precision. We showed in this article how to have a precise estimation of the fluctuation using Monte-Carlo control tests for each fixed set of parameters when theoretical calculations are not available and that Observed Fisher Information Matrix was able to evaluate an upper bound of the order of magnitude of those fluctuations. We were able, for instance, to foresee by such verifications that applying MLR to reconstruct budding sites geometries would yield meaningless estimated parameters. Indeed, due to the small number of sampled positions on the growing bud, and to a stronger positioning uncertainty due to stronger backward noise, random fluctuations dominate in our simulation tests. In such cases, improvements to the data such as 3D localizations or reduced positioning uncertainty $\bar{\sigma }$ would be required to make further progress (see details in supplementary information S1 File). Fortunately, technological advances in the field continue to improve image quality, and will allow MLR to become more powerful in the future. Overall, maximum likelihood reconstruction applied to STORM and (f)PALM data appears a promising method to obtain quantitative measurements on structures, especially those showing variability.

## Supporting information

S1 File Supplementary informations. This file contains all the supplementary information.
(PDF)

S1 Fig The axial projection of uniform density on a truncated sphere. The three value domains of the indicator function (left) for the plane projection of a constant density laying on the incomplete spherical shell (parameters: R, *θ*—right in black) parallel to a given projection axis (parameter: *ϕ*—right in blue dashed arrow). Remarkable points (empty dots) and their projections (filled dots) are shown: mass center (green), sphere center (black), and border center (red). Note that the projection may change the distance measured between them. The expression of the indicator function is deduced by combining the indicator functions of the following regions: the circle that is the projected edge of the sphere (dashed blue—left) and the ellipse (dashed red—left) that is the projection of the border and the strait line that links their tangency points (dashed blue—left).
(PDF)

S2 Fig Ensemble measurement—distribution of simulated data. The distribution reconstruction procedure used to build the experimental distribution of radii and distribution is applied to simulated data: each simulated set contribute with a gaussian centered on the MLR-estimate and whose variance is given by the inverse of the observed Fisher information matrix (red solid line). The original simulated points produced the histogram. Bins size has been chosen to point out the distribution fluctuation to compare with the reconstruction.
(PDF)

S3 Fig Distortions of various completions distribution. Subset of the simulated particles were selected to see how various localized distribution are distorted in the estimation process. The shape of the distribution obtained from real data is not consistent with the full completion of all the object neither with a uniform distribution on the interval.
(PDF)

S4 Fig Characterization of the effective error compared to the estimated one. **(a) Distribution of the normalized error on radii**, Actual errors are normalized by the std. estimated from inverse observed Fisher information matrix. **(b) Distribution of the normalized error on completion**. Actual errors are normalized by the std. estimated from inverse observed Fisher information matrix. **(c) Actual versus estimated error on radii.** Blue dashed lines: estimated std. equal to actual error. **(d) Actual versus estimated error on completion** Blue dashed lines: estimated std. equal to actual error.
(PDF)

S5 FigIll-formed particle of 1550 measured proteins with estimated size equal to 90nm.The PALM data localized positions (*x*_*i*_, *y*_*i*_) (orange dots) are shown superimposed on the probability density for the spatial positions of the localized proteins according to the estimated precision of positioning ($\sum_{i}e^{\|\vec{r}-{\vec{r}}_{i}{\|}^{2}/2\sigma_{i}^{2}}/2\pi \sigma_{i}^{2}$ –color and iso-density lines). The red circles and arrows display the putative positions of two aggregates stuck together that may explain the elongated structure.(PDF)

S6 FigReconstruction error of incomplete spheres fitted by complete spheres.The error between original *R* = 65 nm and reconstructed radius is shown as function of the orientation *ϕ* and the completion *θ* of the original particles simulated. *N*_*s*_ = 400 particles were simulated homogeneously on the interval, all with a actual radius of R = 65 nm and N = 1500 sampled positions. Bias is calculated by a moving average over a disc of $\frac{\pi }{12}$ radius -sampling density affect mostly the dispersion and only weakly the bias.(PDF)

S7 Fig Correlation of radius error with the error on the estimated localization accuracy. (PDF)

S8 Fig Example of four experimental image of HIV1-virus like particles. (PDF)

S9 Fig Sampling size dependence of the precision on the reconstructed radius. Each cross gives the dispersion of the estimated radii of a cohort of complete spheres around the actual radius value for various density of sampled positions.
(PDF)

S10 FigKolmogorov-Smirnov Goodness of fit.*(left)* Distribution of normalized $KS=\sqrt{N}\text{max}\left|{\text{CDF}}_{\text{model}}-{\text{CDF}}_{\text{empirical}}\right|$ values for complete sphere reconstruction of complete spheres *(blue)*, and truncated spheres. *(red)*. *(right)* *KS* distribution mean value (colors and isocontours) for complete sphere reconstruction of simulated truncated spheres as function of initial completion *θ* and orientation *ϕ*. The red dashed area shows the region for which 90% of the estimated structure are rejected by the the Kolmogorov-Smirnov test for a normalized *KS* value of 1.3.(PDF)

S11 Fig Lateral sample drift correction using Au fiducials. Due their stability, Au fiducials can easily be separated from VLPs within the sample. Shown are two examples from independent experiments (*A, B*). (*1*) Individual fiducials were selected (uncorrected), (*2*) their *X* and *Y* position tracked over the full length of the movie (left column) and (*3*) fitted with a polynomial function (*red line, left column*). The fitting function is then used to (*4*) correct all localizations within the sample including the Au fiducial (drift corrected). The accuracy of the fitting (RMSE) was for *A*: *x* = 16.7*nm*, *y* = 18.4*nm* and for *B*: *x* = 25.1*nm*, *y* = 26.6*nm*. The accuracy corresponds to the lateral localization spread of the drift corrected particle and includes the localization precision.
(PDF)

S12 Fig Theoretically determined localization precision. Due to the low number of switching cycles (blinks, on average 3.5) of mEos2 in our experiments (*A*), an experimental determination of the localization precision is not accurate. Hence, we rely on the theoretical calculation as presented in (Thomson et al., *Biophysical Journal*, **2002** May 31;82(5):2775–83). We find an average localization precision of *σ* = 17.6*nm* for single localizations (*B*) and *σ* = 13.4*nm* for grouped localizations (*B*). Shown data from three independent experiments.
(PDF)
