# Supplementary material for: Single particle maximum likelihood reconstruction from superresolution microscopy images
Source: PLoS One. 2017 Mar 2;12(3):e0172943. doi: 10.1371/journal.pone.0172943 (PMC5416903; doi:10.1371/journal.pone.0172943)
Supplement: S1 File — This file contains all the supplementary information. (PDF) [file pone.0172943.s001.pdf]

Supplementary Information for Single Particle Maximum Likelihood Reconstruction from Superresolution Microscopy Images

Timothée Verdier,[1] Julia Gunzenhauser,[2] Suliana Manley,[2] and Martin Castelnovo[1]

[1)] *Laboratoire de Physique, ENS Lyon, Lyon, France*

[2)] *Laboratory of Experimental Biophysics, EPFL, Lausanne, Swizterland*

I. CALCULATION SUPPLEMENTS

A. Center and orient image using geometrical moments

We used the geometrical moments of the point distribution to center and orient it in the focal plan. We consider here the N superresolution measured positions: $\{\vec{r_j} = (x_j, y_j)\}$. The actual label positions $\vec{X_i}$ are assumed to be equal to the measured position up to a Gaussian noise:

$$\vec{r_i} = \vec{X_i} + \vec{\sigma_i} \tag{1}$$

The position of the center of mass of the image is given by the first order moment:

$$\vec{r}_{CM} = \frac{1}{N} \sum_j \vec{r}_j \tag{2}$$

If the number of localized (sampled) positions N is large enough, the distribution mass center is quasi-invariant under the Gaussian convolution, thanks to the isotropy of the latter and the statistical variations follows a central limit theorem:

$$\mathrm{std}_g(\vec{r}_{CM}) \propto \sqrt{\frac{\sum_j \sigma_j^2}{N^2}} = \frac{\bar{\sigma}}{\sqrt{N}} \tag{3}$$

Where the variations is evaluated over the possible positioning error realization $\{\vec{g_i}\}$. According to central limit theorem, the center of mass of the sampled positions furthermore converges towards the center of mass of the underlying label density as the number of localized (sampled) positions N increases: the typical fluctuations around this mean value are of order $R/\sqrt{N}$ (where R is the radius of the particle). The distribution mass center is therefore a very good choice as origin. Centering each distribution on its projected center of mass removes two degrees of freedom (the distribution centers coordinates (x_c, y_c)).

Once the distribution is centered, we look for the symmetry axis of the projected distribution. In a reference basis with axis aligned along the symmetry axis of the distribution, the second order moment matrix is diagonal. We use this property to find the rotation between the current basis and the reference one. We diagonalize the real symmetric matrix of second order moments:

$$M = \frac{1}{N-1} \sum_j \begin{pmatrix} x_j^2 & x_j y_j \\ y_j x_j & y_j^2 \end{pmatrix} = \begin{pmatrix} \cos\alpha & -\sin\alpha \\ \sin\alpha & \cos\alpha \end{pmatrix} \begin{pmatrix} \lambda_- & 0 \\ 0 & \lambda_+ \end{pmatrix} \begin{pmatrix} \cos\alpha & -\sin\alpha \\ \sin\alpha & \cos\alpha \end{pmatrix}^t \tag{4}$$

where the angle α is the angle of principal axis with respect to the original orientation of the projected structure, while the λs are the eigenvalues of the second order moments matrix M. Reference orientation is chosen such that $\lambda_- \leq \lambda_+$, and thus the orientation is deduced up to angle $[\pi]$. In the case of a truncated sphere this constraint places the symmetry axis along the x-axis. However the orientation angle is only defined $[\pi]$ this way (eigenvectors are only defined up to a minus sign).

$$M = \begin{pmatrix} a & c \\ c & b \end{pmatrix}$$

$$\alpha(a,b,c)[\pi] = \begin{cases} \operatorname{atan2}(a-b+\sqrt{(a-b)^2+4c^2}, -2c) & c \neq 0 \\ \frac{\pi}{2} & c = 0,\ a > b \\ 0 & c = 0,\ a \leq b \end{cases} \quad (5)$$

Unlike the distribution mass center, the second order moment matrix of the point distribution can significantly been altered by positioning uncertainty modeled by the addition of a Gaussian noise. Indeed position independent noise adds its contribution to the variance. On average we have:

$$\langle M_{vecr} \rangle_g = M_{\vec{X}} + \mathrm{Cov}_g \quad (6)$$

In contrast, the orientation given by the second order moments matrix are robust to an isotropic measurement uncertainty. The covariance matrix for isotropic noise (Cov_g) is a multiple of identity, therefore the resulting matrix remains diagonal in the same basis as the original one an the aforementioned orientation is preserved on average. Fluctuations around the mean are also controlled by the central limit theorem when noise moment exist a least up to fourth order (a condition satisfied by the Gaussian model). The covariance of the matrix coefficient is thus also $\propto 1/N$: under the condition that the number of localized position N is large enough, the noise only yield small fluctuations of the matrix coefficients around their mean values. When this assumption is valid we can use the error propagation to link the variance of the eigen-vectors orientation to the variances of the point distribution caused by the positioning error:

$$\begin{aligned} \operatorname{var} \alpha = & \; (\vec{\nabla}_{(a,b,c)}\alpha)^t \mathrm{Cov}(a,b,c)(\vec{\nabla}_{(a,b,c)}\alpha) \\ \overset{c \ll a \leq b}{=} & \; \frac{\operatorname{var}(c)}{(b-a)^2} \end{aligned} \quad (7)$$

Direct evaluation of $\mathrm{var}\, c$ yield to an expression involving fourth and second order moments of noise and localized position distribution. For Gaussian and spherical distribution, fourth order moments scale as the square of the second order moments:

$$\mathrm{var}\, c \overset{c \ll a \leq b}{\simeq} \frac{\lambda_- \lambda_+}{N} \tag{8}$$

As $c \ll 1$, we have $a \simeq \lambda_-$ and $b \simeq \lambda_+$ This gives the α error bar for each particle:

$$\mathrm{var}\, \alpha \propto \frac{\lambda_+ \lambda_-}{N(\lambda_+ - \lambda_2)^2} \tag{9}$$

This expression gives the accuracy in the determination of a reference orientation for the positions distribution using 5 and yield an confidence interval $\Delta\alpha = \sqrt{\mathrm{var}\, \alpha}$, including the effect of an isotropic noise and finite sampling ($N \gg 1$). Anisotropy of the original position distribution guarantees $\lambda_+ - \lambda_- \gg 1$ whereas position uncertainty contribute by an additive constant in λ_+ and λ_-. Thus the more isotropic the original position distribution, the less robust the orientation regarding positioning error and finite sampling.

To eventually eliminate the α parameter, we need a $[2\pi]$ determination that is an information about the asymmetry of the distribution along x once properly oriented. This is done by computing the sign of third order central moments $\mu_{x,y,y}$ in these oriented coordinates and apply a symmetry relatively to the x-axis according to it:

$$\begin{cases} \sum_{i,j,k} x_i . y_i^2 > 0 \; \Rightarrow \; \alpha \to \alpha + \pi \\ \sum_{i,j,k} x_i . y_i^2 < 0 \; \Rightarrow \; \alpha \to \alpha \end{cases} \tag{10}$$

Centering and orientation using geometrical moments leads to the simplified problem of fitting three degrees of freedom, namely the radius, the completion and the tilt of the particle $\vec{\beta} = (\mathrm{R}, \theta, \phi)$.

B. Computation of the projected density of an incomplete spherical shell

We obtained an analytical expression of the planar projection of a constant density on any incomplete spherical shell as the product of the projection of a reference constant density d° and an indicator function. The reference density is obtained by projecting half a sphere along its symmetry axis. From a parametrization of the surface of the form $z = f(x, y)$, the local measure of projected surface is:

$$\mathrm{d}S = \sqrt{1 + \|\vec{\nabla} f\|^2}\, \mathrm{d}x\, \mathrm{d}y \tag{11}$$

Using the obvious parametrization $z(x, y) = \sqrt{R^2 - (x^2 + y^2)}$ of an hemisphere of radius R in Cartesian coordinates, we deduce its projected density:

$$d^\circ(\rho) = \frac{1}{\sqrt{1 - (\rho/\mathrm{R})^2}} \tag{12}$$

Where $\rho = \sqrt{x^2 + y^2}$ is the distance to origin in polar coordinates. It appears that the measure d° diverges close to the boundaries ($\rho = R$) where the surface normal is parallel to the (x, y) plane and the unit surface on the sphere is projected on a line.

The indicator function then traduces the completion: its value at a given point corresponds to how often the projection axis crosses the shell, hence its values are in $\{0, 1, 2\}$ as shown on S1 Fig. And the domain of each of those value are delimited by a circle, an ellipse and a strait line. Respectively, the projected contour of the sphere (Ind_1) centered in x_c, the projected contour of the completion edges (Ind_2) centered in x_e and the line that join their tangency points (Ind_3) of abscissa $x_{tangency}$. Those equation are easy to parametrize in the axis system described in the above section:

$$d_p[\vec{\beta}](x, y) = \frac{1}{\sqrt{1 - ((x - x_c)^2 + y^2)/\mathrm{R}^2}} \times \mathrm{Ind}[\vec{\beta}](x, y) \tag{13}$$

$$\mathrm{Ind}[\vec{\beta}](x, y) = 2.\mathrm{Ind}_1.\mathrm{Ind}_3.(1 - \mathrm{Ind}_2) + \mathrm{Ind}_2 \tag{14}$$

With the indicator functions given by:

$$\begin{aligned}
\mathrm{Ind}_1[\vec{\beta}](x, y) &= \begin{cases} 1 \text{ if } & \|y\| \le \mathrm{R}\sqrt{1 - \frac{(x - x_c)^2}{\mathrm{R}^2}} \\ 0 & \text{else.} \end{cases} \\
\mathrm{Ind}_2[\vec{\beta}](x, y) &= \begin{cases} 1 \text{ if } & \|y\| \le \mathrm{R}\sin\theta\sqrt{1 - \frac{(x - x_e)^2}{\mathrm{R}^2 \sin^2\theta \cos^2\phi}} \\ 0 & \text{else.} \end{cases} \\
\mathrm{Ind}_3[\vec{\beta}](x, y) &= \begin{cases} 1 \text{ if } & x \ge x_{tangency} \\ 0 & \text{else.} \end{cases}
\end{aligned} \tag{15}$$

And the following coordinate for the projected reference points:

$$\begin{aligned}
x_c &= -\mathrm{R}\frac{1 + \cos\theta}{2} \\
x_e &= \mathrm{R}(\cos\theta - \frac{1 + \cos\theta}{2}) \\
x_{tangency} &= -\frac{\mathrm{R}}{8}(-2 + 6\cos\theta + \cos(\theta - 2\phi) + 2\cos 2\phi + \cos(\theta + 2\phi))\csc\phi
\end{aligned} \tag{16}$$

The projected density obtained here $d_p[\vec{\beta}](x, y)$ is then convolved to a Gaussian function of full standard deviation $\bar{\sigma}$ in order to take into account the positioning imprecision of the super-resolution method.

$$g(\vec{X}) = \frac{1}{2\pi\bar{\sigma}} e^{-\frac{\|\vec{X}\|^2}{2\bar{\sigma}^2}} \tag{17}$$

This mathematically regulates the divergence for $\rho \to \mathrm{R}$. But since we perform this step numerically, evaluating the function on grids, we use a regulation procedure. the projected density is not evaluated on a ring of size around $R - \epsilon < \rho < R$ but taken as its mean value over this interval instead:

$$d^{\circ}(\rho) \underset{\mathrm{R}-\epsilon<\rho<\mathrm{R}}{\equiv} \frac{\int_{\mathrm{R}-\epsilon}^{\mathrm{R}} \frac{\rho\,\mathrm{d}\rho\,\mathrm{d}\theta}{\sqrt{1-(\rho/\mathrm{R})^2}}}{\int_{\mathrm{R}-\epsilon}^{\mathrm{R}} \rho\,\mathrm{d}\rho\,\mathrm{d}\theta} = \frac{\sqrt{(2\mathrm{R}-\epsilon)\epsilon}}{\mathrm{R}\epsilon - \epsilon^2/2} \sim \sqrt{\frac{2}{\epsilon \mathrm{R}}} \tag{18}$$

Then the numerical 2D-convolution between the density and the Gaussian g is made using Fast Fourier Transform (FFT) and the relation:

$$d_p * g = \mathrm{FFT}^{-1}(\mathrm{FFT}(d_p).\mathrm{FFT}(g)) \tag{19}$$

when functions are evaluated on a discrete $p_{\times}p$ grid, the direct calculation of N localization likelihood would cost $O(N \times p^2)$ where our approximation using fast Fourier transform costs $O(2p^2 \log p)$ (improved compare to the classic convolution on $p \times p$ grid is in $O(p^4)$) and therefore makes a substantial improvement when $N \geq 2 \log p$, which is guaranteed in practical situations ($N > 10$). Eventually the likelihood values at the localized positions are deduced by linear interpolation from the calculated grid.

II. ESTIMATION OF THE SIMULATED PARTICLE DISTRIBUTIONS

A. Estimated distributions

We used the simulated PALM images to test the efficiency of the MLR procedure used on experimental data in the reconstruction of the distribution of sizes and completion at the level of a population (initial and reconstructed distribution are shown S2 Fig).

The radii of the simulated particles are well retrieved by the procedure and the overall effect of the method is to provide a smoothed image of the exact distribution on a slightly wider interval than the original one (S2 Fig). This comes from the fact that variances of the measure determined with the observed Fisher information matrix are on average larger

than the actually measured error amplitude. This estimation therefore tends to exaggerate the confidence interval of the reconstructed radius. In contrast a sharp peak is visible at $R = 42$ nm, showing that particle can sometimes be associated to a very small estimated variance.

The distribution of simulated particle completions is much less accurate in describing the real distribution fluctuations though the general trend is conserved (S2 Fig). The estimation method is not precise enough to reproduce the details of the actual distribution. We can notice that the region close to full completion ($\theta \simeq \pi$) is depleted on the reconstructed distribution compared to the original one. This comes from both a bias and reconstruction precision that vary over the interval. The effects of this completion dependent error are illustrated in more detail in S3 Fig: a uniform distribution over the reconstruction interval is correctly reconstructed by the method at the exception of a slight depletion on the interval edges, mostly affecting full completion side, and a complementary overestimation at the interval center. Estimations of the completions of actually complete particles are spread over the whole interval (see S3 Fig), explaining this trend. The method also generate a broad distribution for a group of mono-disperse particles peaked around a completion value at the interior of the interval (S3 Fig). However in this case the mode of the estimated distribution still correspond to the average completion of the group. Oppositely, the mode of the reconstructed distribution from the complete particle subpopulation is displaced toward the interval center around $9\pi/10$. This is a strong underestimation yet larger than $4\pi/5$, the value obtained from the experimental data given in the main text. We looked for the minimal range of completion, including fully complete particles, needed to produces a estimated distribution similar to the experimental data -assuming for simplicity uniform distribution over the subdomain. S3 Fig shows that $3\pi/4$ is an appropriate value. This qualitatively support the idea of the presence of incomplete particle in the experimental sample.

III. ESTIMATED VARIANCES

We calculated the observed Fisher information matrix and used its inverse as an estimate of the covariance of the estimation error. The difference between the actual parameters of the simulated particles and the estimated ones, in other words the actual error, normalized

by this estimated values are shown on S4 Fig. The normalized error distribution width is of order 1, showing that the standard deviation obtained this way has the appropriate magnitude. However both distribution are less extended than the reference normal distribution which tels us that our estimations are on average over estimating the magnitude of the errors. S4 Fig describes in more details the correlation between the actual error and the estimated standard deviations. As already pointed out, the estimated standard deviation is larger than the actual error on a vast majority of cases. S4 Fig shows that the actual standard deviation estimate is weakly or not correlated to the actual error and always larger than 2 nm even when the actual error is much smaller. This might indicate that in the present case the sampling of the object forbid a better knowledge of the actual radius even if we might encounter the exact value by chance. A stronger correlation is showed on S4 Fig regarding the completion. Apart for the particle for which the estimation was definitely not consistent (i.e. $|\theta_{\mathrm{simu}} - \theta_{\mathrm{fitted}}| > \pi/6$), the estimated standard deviation is larger than the actual errors (points lay within the higher region delimited by the dashed lines).

IV. ILL-FORMED PARTICLES

Several protein clusters were not included into the experimental data processed for they showed a deformed shape likely associated to aggregations of many forming clusters and obviously did not correspond to the model used to parametrize the VLPs. In the set of particles submitted to the MLR procedure, one was associated to radius much larger than the others. S5 Fig shows the PALM image of this cluster. The scatter representation convention, where each localized tag is displayed by a dot at its most likely position (x_i, y_i), is superposed on the probabilistic representation where the positioning uncertainty is taken into account and each localization is displayed as a Gaussian curve centered on (x_i, y_i) with standard deviation σ_i. The presence of such abnormal VLPs can be explained either as the aggregation of Gag caps, sometimes observed in cryo-electron microscopy or as the anomalous ordering of Gag fused with the fluorescent proteins in the VLP seen as a consequence of the steric hindrance caused by the N-terminus tag (see reference in the main text).

V. RECONSTRUCTION USING A SIMPLE COMPLETE SPHERE MODEL ON SIMULATED IMAGES OF TRUNCATED SPHERES

In this section, we perform simulations of truncated spheres as explained in the main text, but we use the maximum likelihood reconstruction with a complete sphere model (by forcing a completion $\theta = \pi$). This allows us to check whether the reconstruction could be simplified by not disregarding the completion and orientation, which have been shown to be rather poorly determined by the truncated model (see main text). S6 Fig shows the geometry dependent bias between the actual radius $R = 65$ nm, equal for all simulated particle PALM measurement, and the one estimated by the MLR. It is observed that the error on the radius determination is strongly dependent on the value of initial completion and orientation. Not surprisingly, the smallest errors are obtained for almost full completion $\theta \simeq \pi$. However the error can be as high as 5-10 nm for certain values of (θ, ϕ), and is therefore larger than the values obtained by the reconstruction procedure using the truncated sphere model (the bias is $\Delta R < 1$ nm and the dispersion is $\sigma_{\Delta R} \simeq 1.3$ nm compared to 2.9 nm in this case). This shows that although the completion and orientation are determined in a poorer way than the radius, these parameters are still essential in order to get a more accurate determination

of the radius when incompletion is suspected.

VI. CORRELATION OF THE ERROR OF RECONSTRUCTED RADIUS WITH THE ACCURACY OF SINGLE PROTEIN LOCALIZATION EVENT

The method can be seen as a deconvolution treatment whose results depend on the correctness of the location accuracy estimated during the PALM treatment. Convolution by a larger uncertainty spreads out the point distribution and is therefore expected to increase the effective scale at which the object appears. The following additionnal test was ran in order to quantify the effect of an underestimation/overestimation of the positionning accuracy: ($N_s = 400$) spheres where simulated with ($N = 1500$ localizations) a fixed radius of $R = 65nm$ using the method detailed in the article (each point accuracy was randomly drawn from a gaussian distribution of mean $20nm$ and std $5nm$), but the accuracy passed to the algorithm were intentionally biased with an accuracy error $\Delta\sigma$. Radii where then recovered using the maximum likelihood method. As expected, the fitted radii distribution is displaced to compensate the bias: a global underestimation of $\Delta\sigma = -3nm$ on the accuracy generate an overestimation of $\Delta R = 3nm$ on average. (N.B. A closer look at the fitted radii distribution shows that its dispersion is not completely symmetric in $\Delta\sigma$ especially for $\Delta\sigma = -10nm$. This can be caused by the way we treated the absurd situation arising when a position is was reported to be very precisely measured at first (say $\sigma < 10nm$), for which bias lead to meaningless null or negative accuracy estimate passed to the algorithm.

VII. TYPICAL IMAGES FROM EXPERIMENTS

S8 Fig shows typical images from experiments, that are analyzed using our MLR procedure.

VIII. SAMPLE SIZE DEPENDENCE OF THE PRECISION ON THE RADIUS DETERMINATION

As expected from the law of large numbers, the accuracy in R (std R_{fitted}) will asymptotically scales like $\sigma/\sqrt{N}$. This is shown on S9 Fig.

IX. ESTIMATING THE GOODNESS OF FIT OF THE RECONSTRUCTION PROCEDURE

The strong difference observed between the estimated effective complete-sphere radius on one hand and the actual radius of the truncated sphere on the other hand raise the general question of testing the significance of the procedure estimations. Beyond determining the optimal parameters obtained through the reconstruction process, is it possible to address the quality of the fitting? We suggest to use a 2D generalization of Kolmogorov-Smirnov statistical test in order to perform such an estimation. This approach tests a statistical deviation between the model cumulative distribution and the empirical cumulative distributions obtained from the measured position. In our case, this would require the comparison of 2D spatial distribution of protein localization with the theoretical spatial distribution generated from the model with the parameters determined by the reconstruction procedure. The general principle of distribution comparison found by Kolmogorov and Smirnov in 1D is to compute the cumulative distribution value in both cases, and define the largest gap between the two functions as the test parameter, also known as the normalized KS value:

$$KS = \sqrt{N}\max_X | \int_0^X d[\vec{\beta}_*](x)\mathrm{d}x - \frac{1}{N}\sum_{x_i<X} 1| \tag{20}$$

In 1D, for a sample of N points randomly drawn from a reference distribution with continuous density $d[\vec{\beta}_*]$, the test parameter KS is then distributed asymptotically for large systems according to the so-called Kolmogorov distribution. As a consequence, a statistical test on the KS value determine whether the hypothesis that the sample is randomly drawn from

the distribution can be accepted given a threshold uncertainty or risk. In 2D, it is more difficult to define properly and in a unique way the cumulative distribution function, as three independent definitions of the cumulated distribution are possible following permutations of the sens of integration in each dimension. In this case, Peacock suggested the following method in order to estimate the KS: find the largest gap between cumulative distributions for all independent ways to compute cumulative distributions (for instance $(x > X, y > Y), (x > X, y < Y), (x < X, y > Y)$), and then use the maximum value among these largest gap as KS. This yield a value independent of the chosen orientation of the space. In 2D, however the test statistical values are not anymore distribution independent and must be evaluated on a case by case basis.

The distribution of such KS values are shown in S10 Fig for our maximum likelihood approach: using the parameter values corresponding to the maximum likelihood of a complete sphere model, we estimated the best theoretical spatial distribution of protein corresponding to simulated images. We then compared this fits to the experimental distribution of measured position (the image), thanks to their cumulative distributions. The largest gap between cumulative distributions giving the KS value. We simulated two different group of particle. In the first group, all the particle are fully complete spheres. This group give the reference of the KS purely stochastic fluctuations expected from the finite sampling effect. On the contrary, when measures of truncated spheres are fitted with the complete sphere model, larger KS values are seen indicating that the measured position deviate in a larger part from the fitted model: the reconstruction cannot be considered as satisfactory.

An arbitrary limit of the normalized KS value is chosen in order to retain the largest part of the reference distribution (limit the false rejection - the risk value) and reject a much as possible of the non complete spheres (limit the false positive). The power of the test to discriminate between the complete and truncated structure depends on the sampling value. S10 Fig emphasizes this effect for an arbitrary value of $KS \simeq 1.3$ (the red dashed area correspond to a rejection rate of at least 90% of the tested fits) and various sampling ($N = 1500$,$N = 5000$). The mean of the distribution of normalized KS values as a function of the initial completion and orientation of the spheres used in the simulation). Obviously, structure with the closest geometry require denser sampling to be distinguished (almost complete spheres and half sphere viewed from the top cannot be differentiated from complete spheres).

These results show that the Kolmogorov-Smirnov statistical test can be used in order to address the goodness of fit issue after our reconstruction procedure.

X. 3D MEASUREMENT SETUPS COMPLEMENT: SPATIALLY INHOMOGENEOUS POSITIONING PRECISION

As emphasized in the main text conclusion, a promising improvement would be to use data with 3D positions to avoid the loss of information inherent to the projection. However depending on the solution chosen to implement the 3D imaging, the positioning precision is likely to be inhomogeneous with depth in addition to the effects originating from the TIRF configuration already discussed in the main text. This effect has been investigated theoretically by simulating the optical devices (see reference in main text). In both bi-plane and astigmatic setup, the expected fluctuations of the positioning precisions are typically lower than the variation of photon yield by the inhomogeneous TIRF excitation field or the fluorophore stochastic emission.

In contrast, in the most commonly used astigmatic setup, the deformation of the point spread function give rise to an anisotropic localization precision in the (x, y) plane, thus the overall shape of the image is deformed in the plane compare to the original object which impairs the orientation efficiency of the moments method used here. The bi-plane imaging method has the advantage to keep a theoretical precision isotropic in (x, y) and relatively constant with depth within the imaging volume of depth 600 nm chosen for the simulation in the cited reference.

However, considering the reverse problem of estimating the best structure match for a given image, the positioning precision is directly estimated from the measurements. So it is considered as known in this work and we assume also that it can be estimated correctly enough (see section VI otherwise). Spatially inhomogeneous positioning error should primarily impact the orientation of particle found by the moment method. Then the validity of the average positioning error approximation we used can be limited by the evolution of the positioning error with depth. An important variation of positioning error with depth would demand to take into account each point positioning error. This would require much longer computation but the likelihood method exposed in this work would remains valid. This is then on the side of the superresolution data treatment that limitations may arise preventing

a correct estimation of the positioning uncertainty.
