# Supplementary figures and images for: Single particle maximum likelihood reconstruction from superresolution microscopy images
Source: PLoS One. 2017 Mar 2;12(3):e0172943. doi: 10.1371/journal.pone.0172943 (PMC5416903; doi:10.1371/journal.pone.0172943)

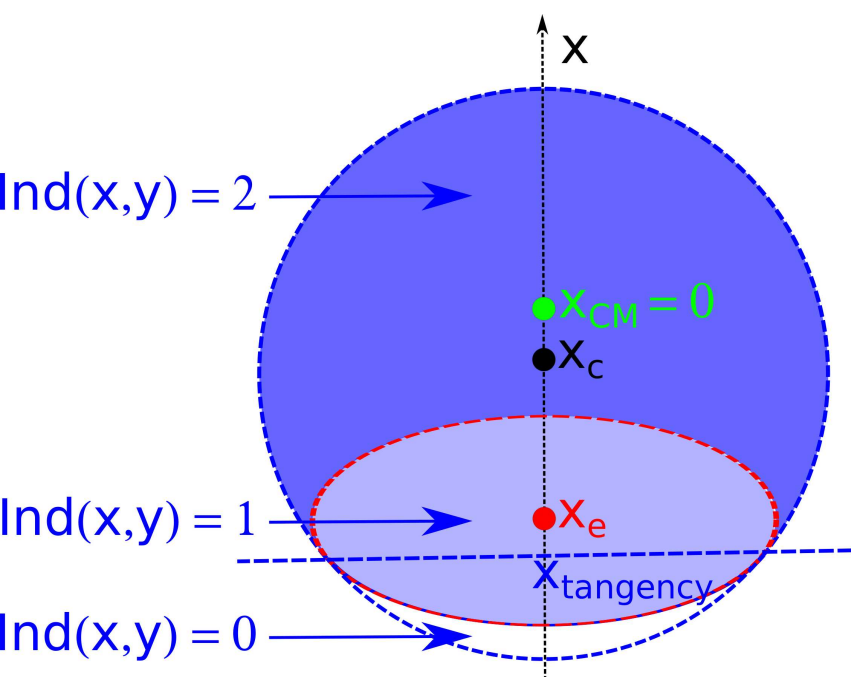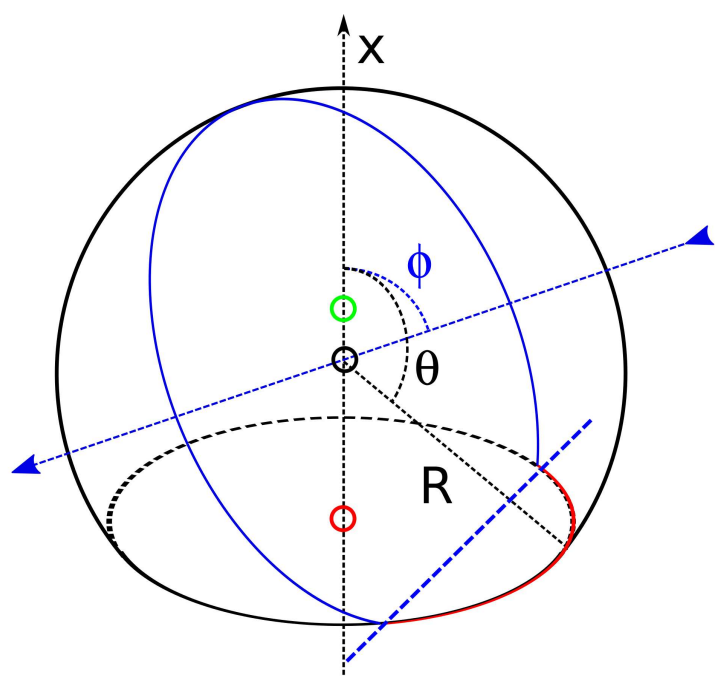

Supplement: S1 Fig — The three value domains of the indicator function (left) for the plane projection of a constant density laying on the incomplete spherical shell (parameters: R, θ—right in black) parallel to a given projection axis (parameter: ϕ—right in blue dashed arrow). Remarkable points (empty dots) and their projections (filled dots) are shown: mass center (green), sphere center (black), and border center (red). Note that the projection may change the distance measured between them. The expression of the indicator function is deduced by combining the indicator functions of the following regions: the circle that is the projected edge of the sphere (dashed blue—left) and the ellipse (dashed red—left) that is the projection of the border and the strait line that links their tangency points (dashed blue—left). (PDF) [file pone.0172943.s002.pdf]

(a)

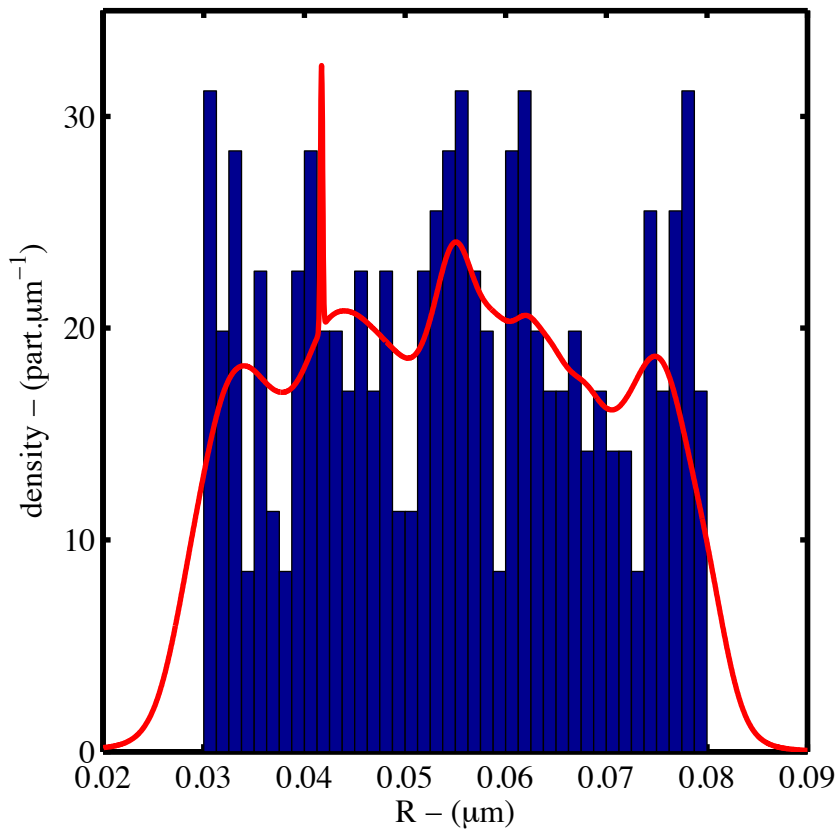

(b)

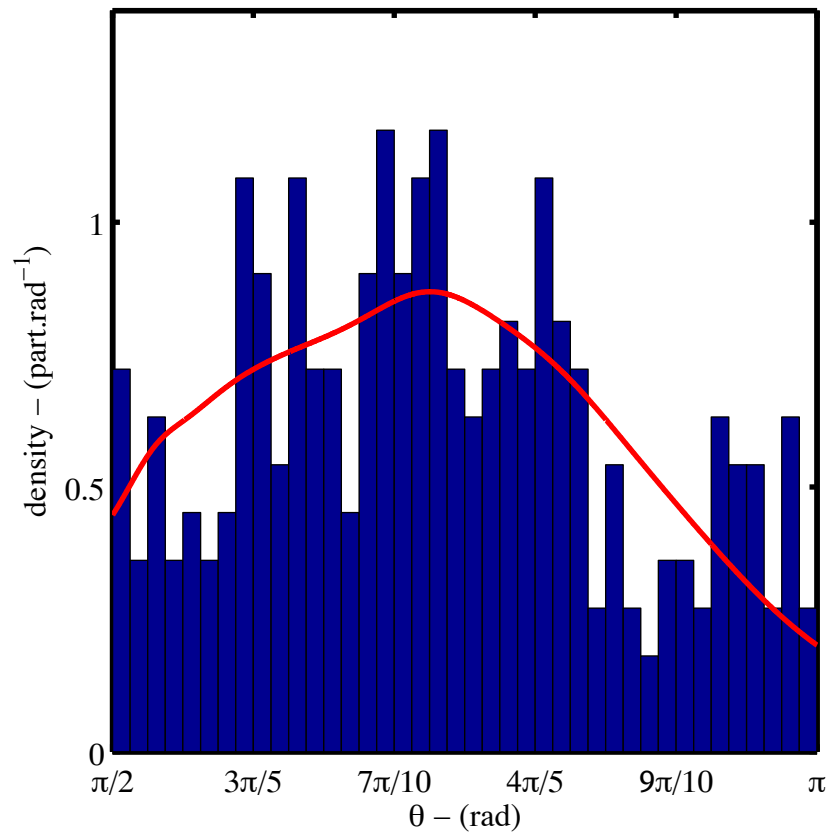

Supplement: S2 Fig — The distribution reconstruction procedure used to build the experimental distribution of radii and distribution is applied to simulated data: each simulated set contribute with a gaussian centered on the MLR-estimate and whose variance is given by the inverse of the observed Fisher information matrix (red solid line). The original simulated points produced the histogram. Bins size has been chosen to point out the distribution fluctuation to compare with the reconstruction. (PDF) [file pone.0172943.s003.pdf]

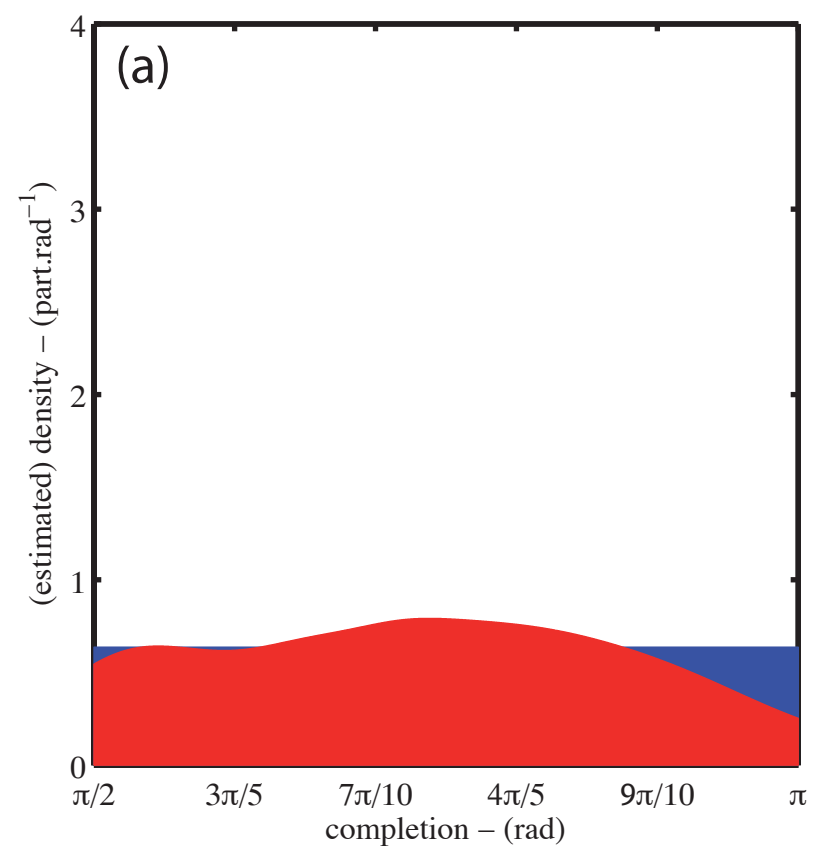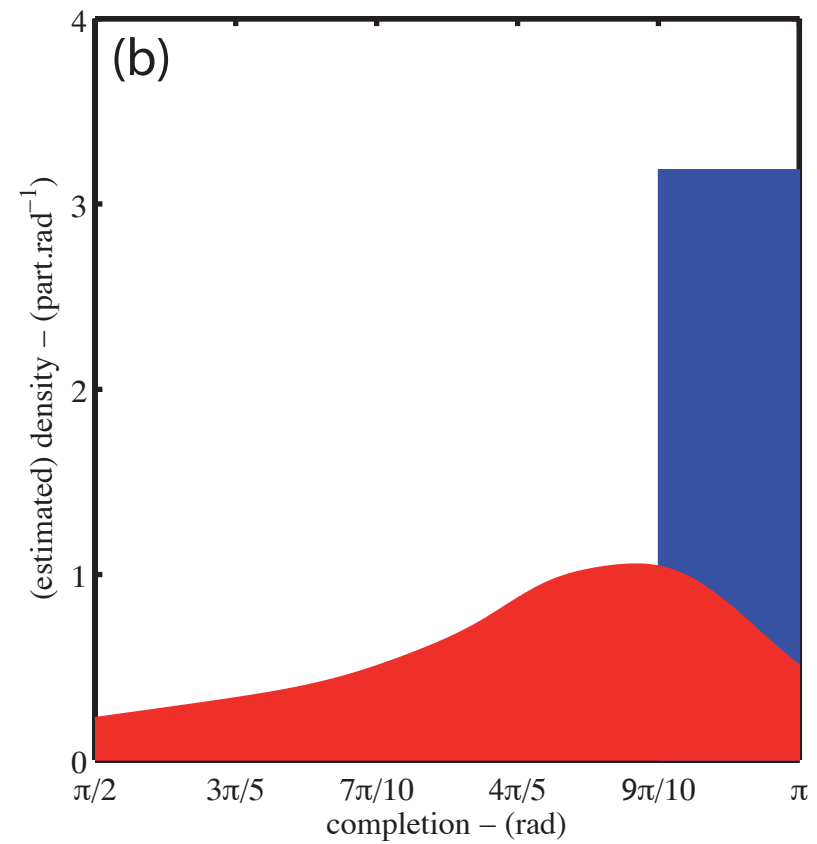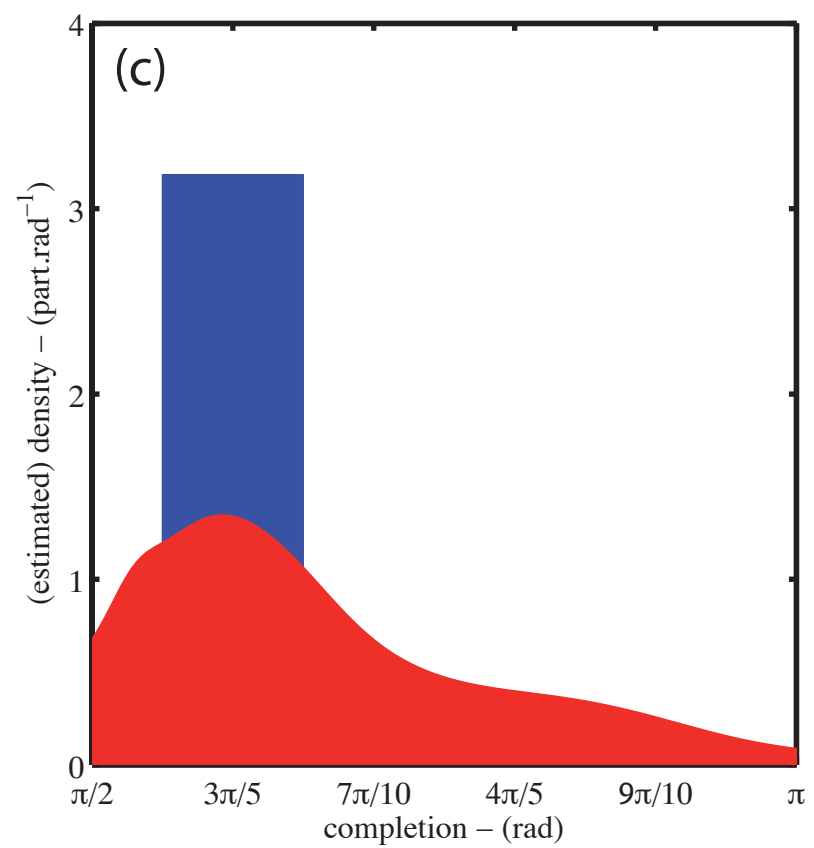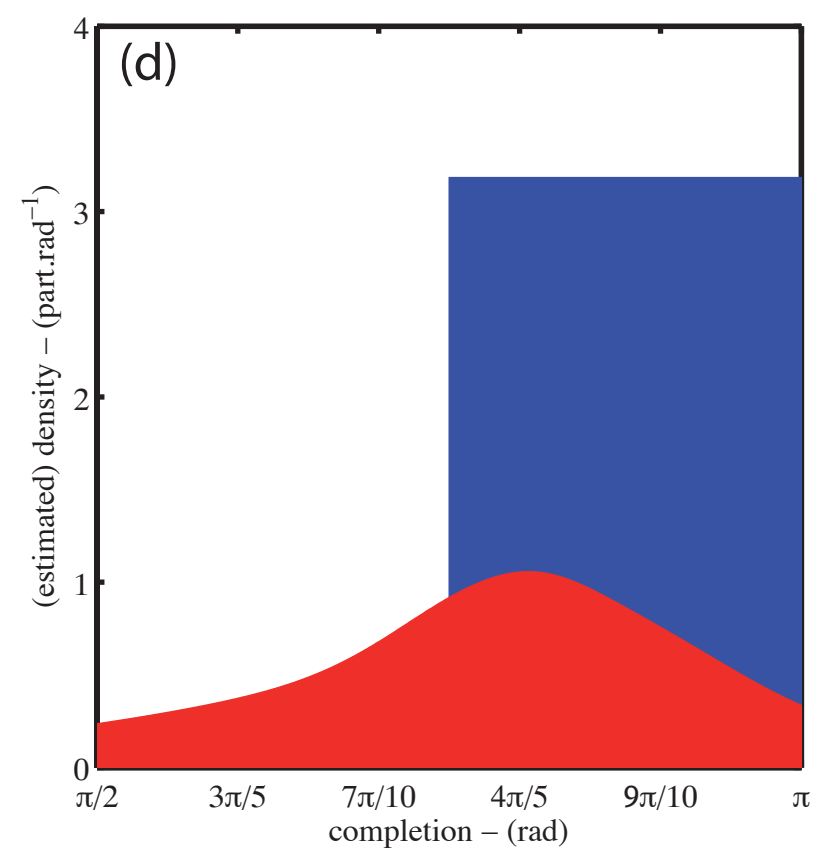

Supplement: S3 Fig — Subset of the simulated particles were selected to see how various localized distribution are distorted in the estimation process. The shape of the distribution obtained from real data is not consistent with the full completion of all the object neither with a uniform distribution on the interval. (PDF) [file pone.0172943.s004.pdf]

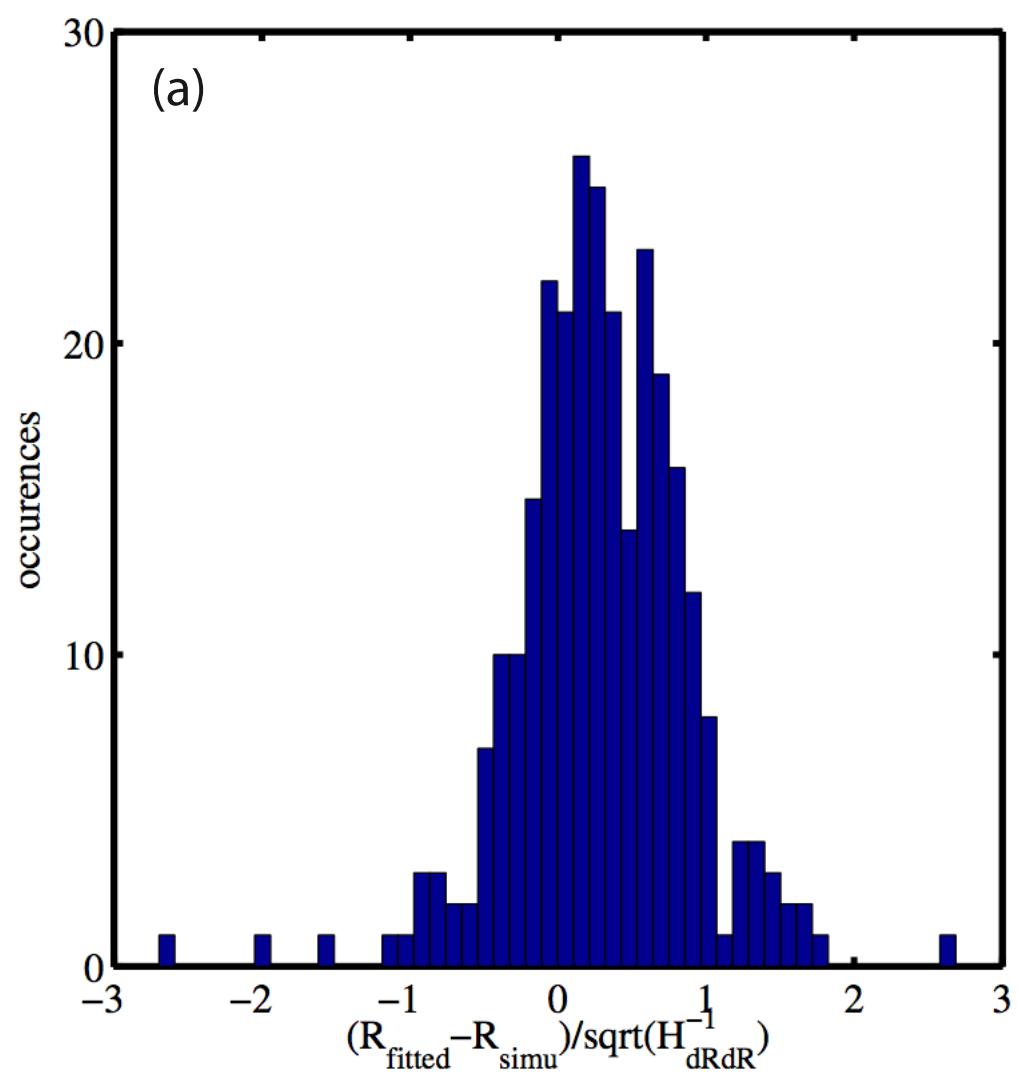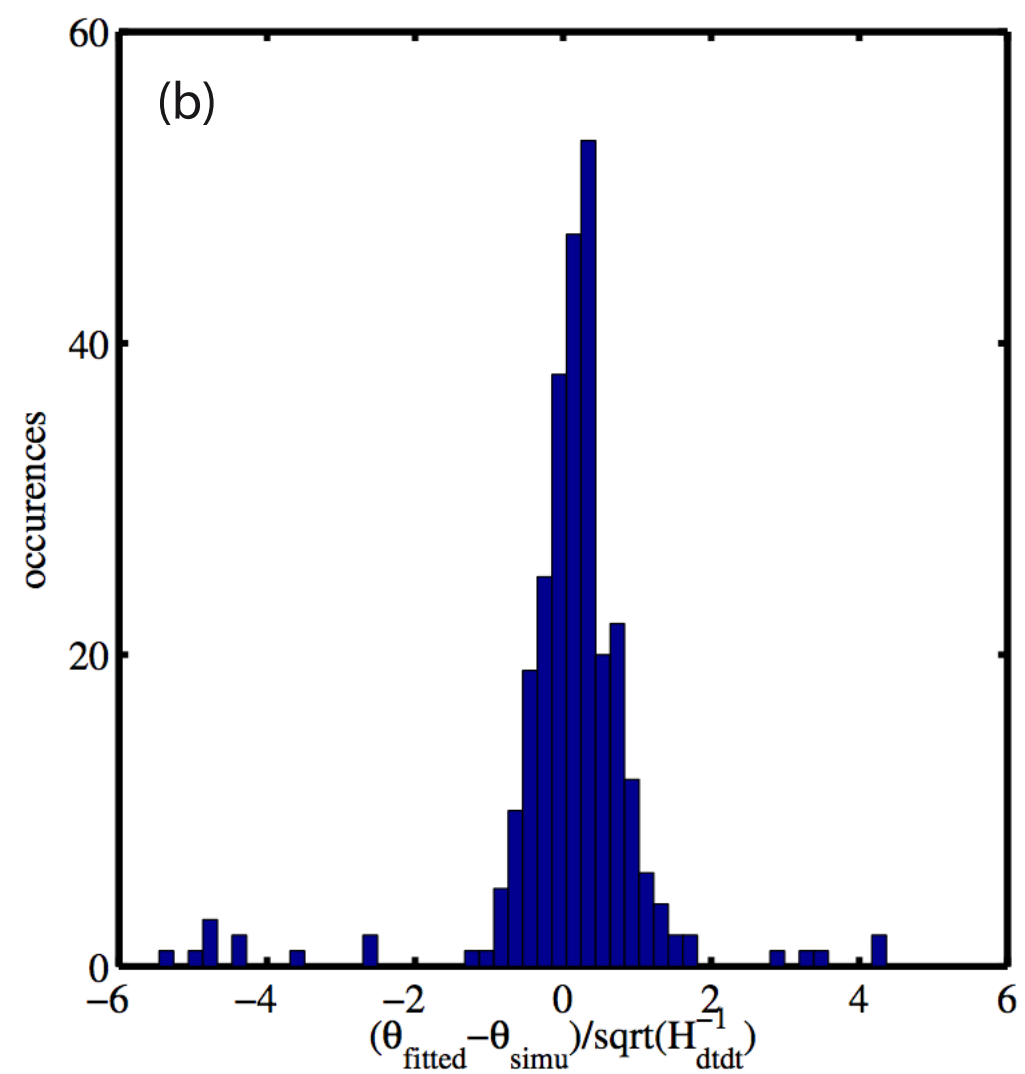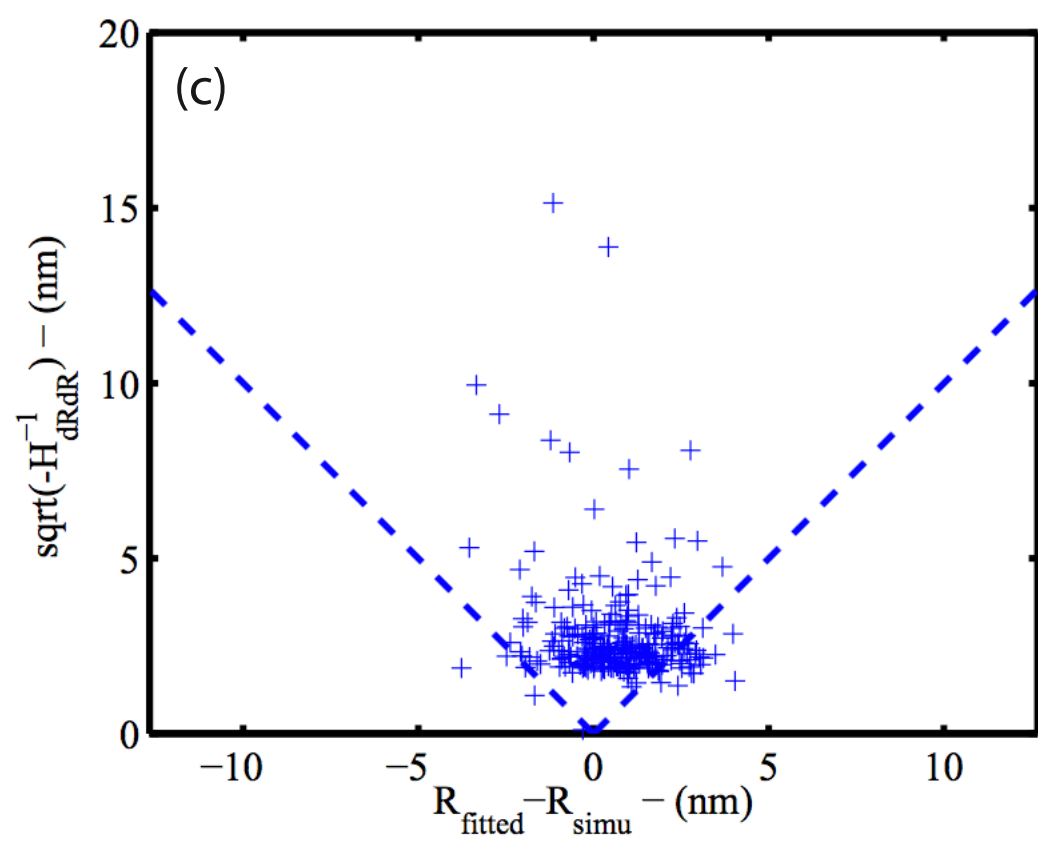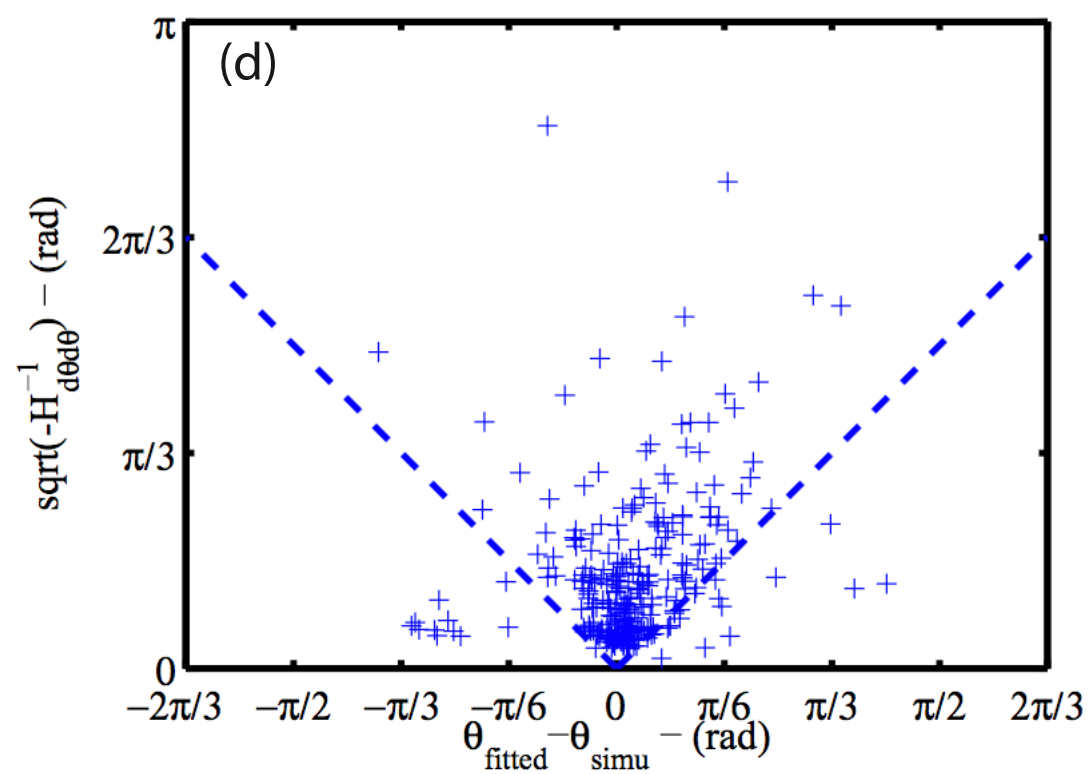

Supplement: S4 Fig — (a) Distribution of the normalized error on radii, Actual errors are normalized by the std. estimated from inverse observed Fisher information matrix. (b) Distribution of the normalized error on completion. Actual errors are normalized by the std. estimated from inverse observed Fisher information matrix. (c) Actual versus estimated error on radii. Blue dashed lines: estimated std. equal to actual error. (d) Actual versus estimated error on completion Blue dashed lines: estimated std. equal to actual error. (PDF) [file pone.0172943.s005.pdf]

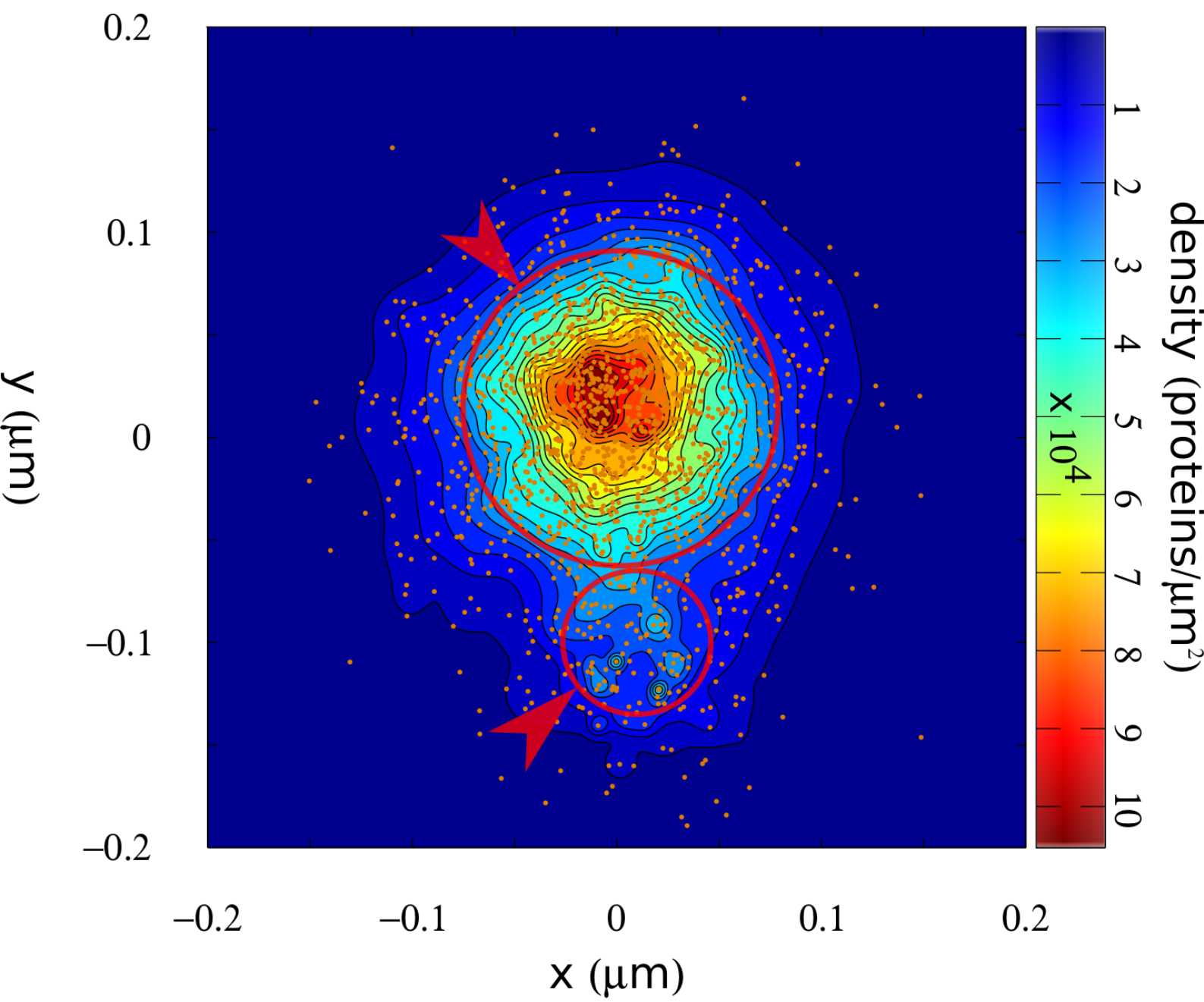

Supplement: S5 Fig — The PALM data localized positions (xi, yi) (orange dots) are shown superimposed on the probability density for the spatial positions of the localized proteins according to the estimated precision of positioning (∑ie‖r→-r→i‖2/2σi2/2πσi2 –color and iso-density lines). The red circles and arrows display the putative positions of two aggregates stuck together that may explain the elongated structure. (PDF) [file pone.0172943.s006.pdf]

$R_{\text{fitted}}$  error nm

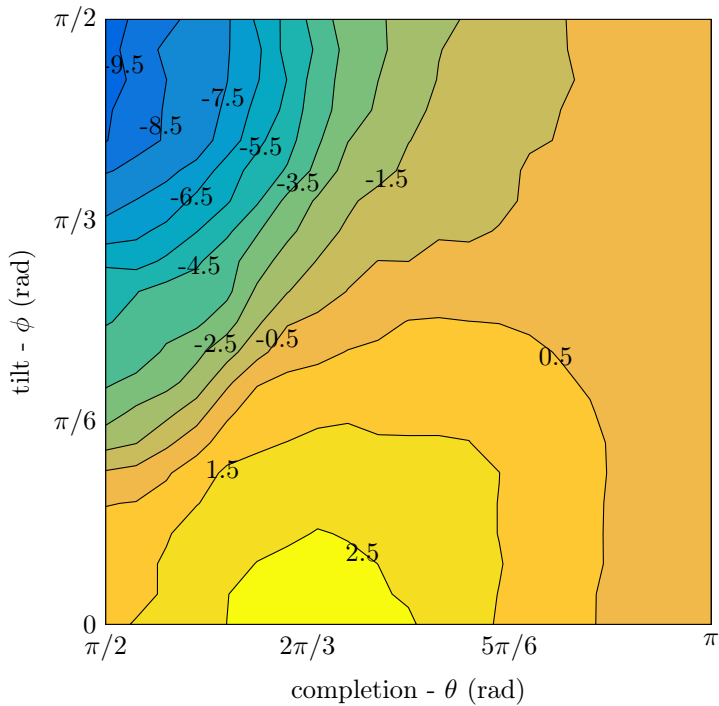

Supplement: S6 Fig — The error between original R = 65 nm and reconstructed radius is shown as function of the orientation ϕ and the completion θ of the original particles simulated. Ns = 400 particles were simulated homogeneously on the interval, all with a actual radius of R = 65 nm and N = 1500 sampled positions. Bias is calculated by a moving average over a disc of π12 radius -sampling density affect mostly the dispersion and only weakly the bias. (PDF) [file pone.0172943.s007.pdf]

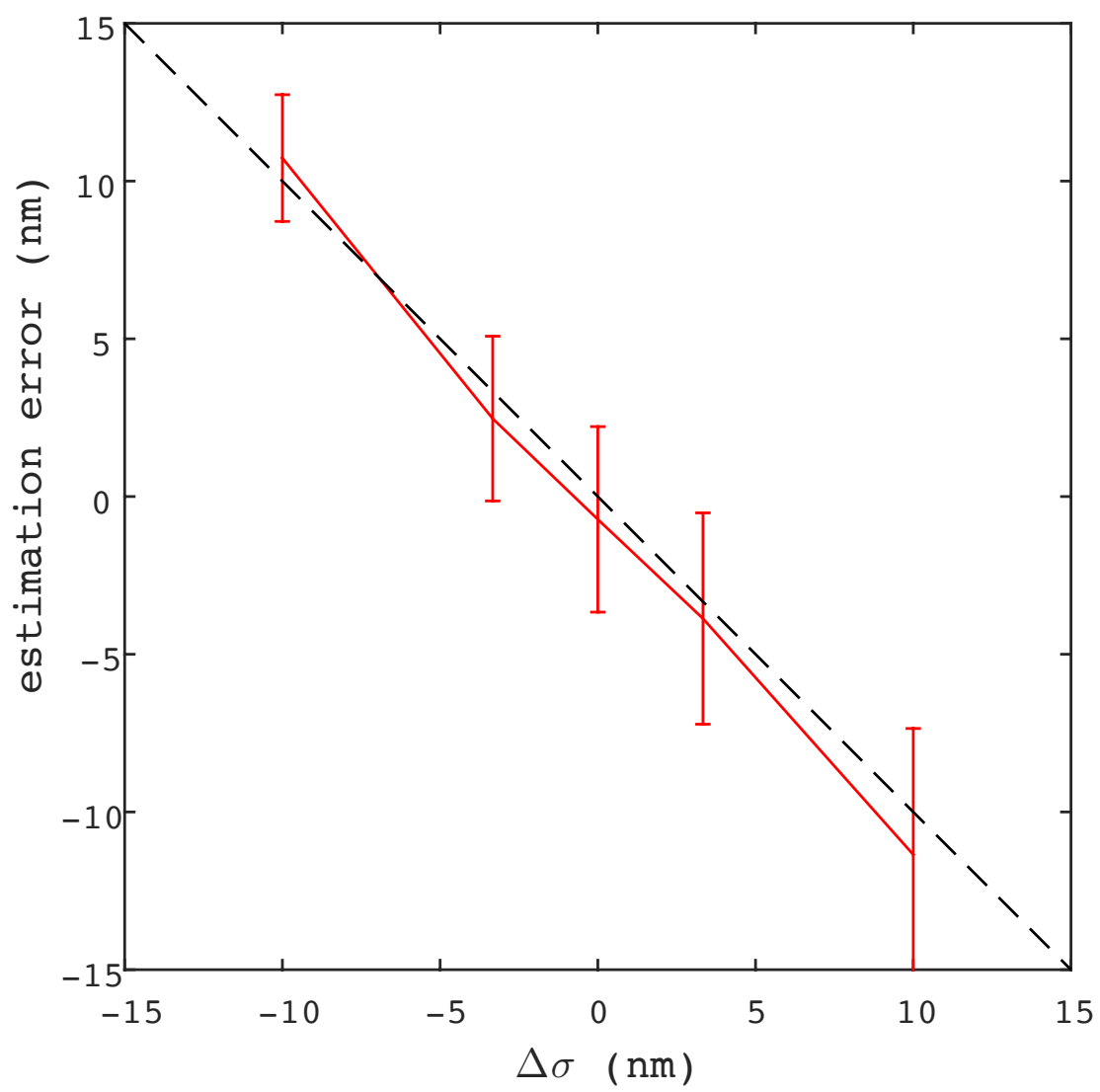

Supplement: S7 Fig — (PDF) [file pone.0172943.s008.pdf]

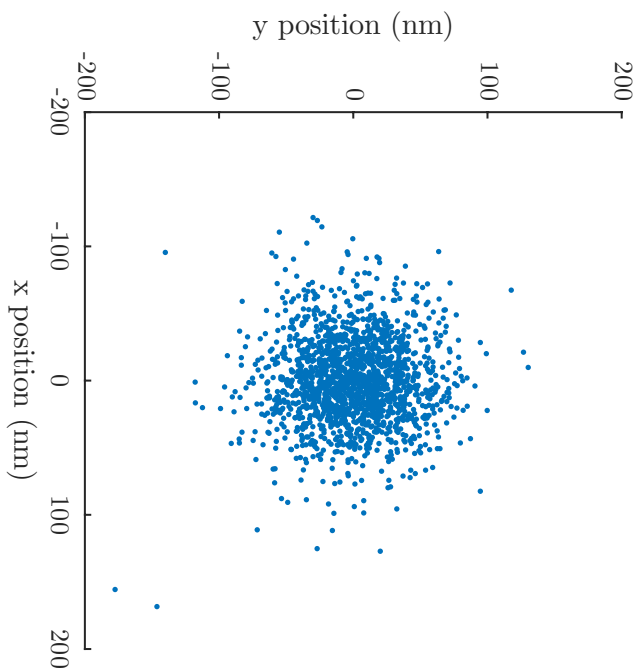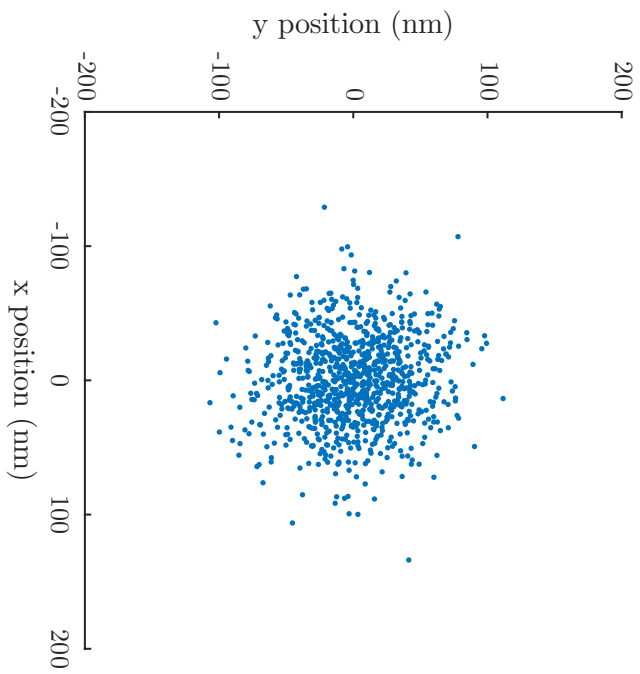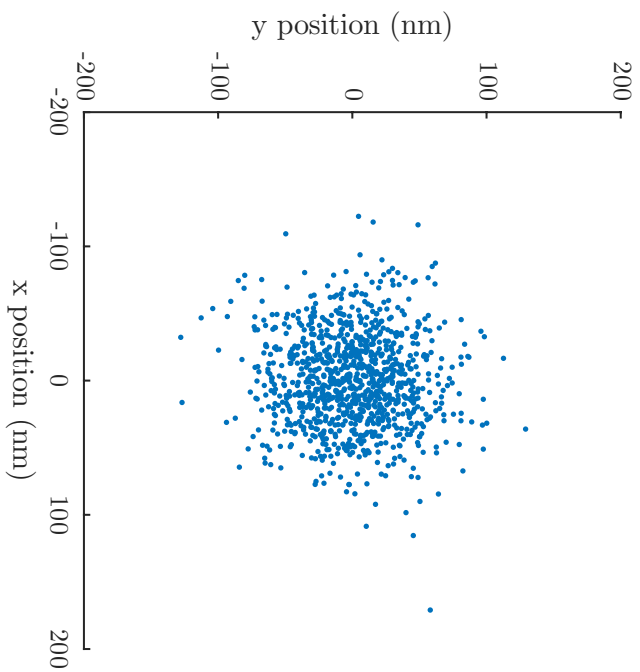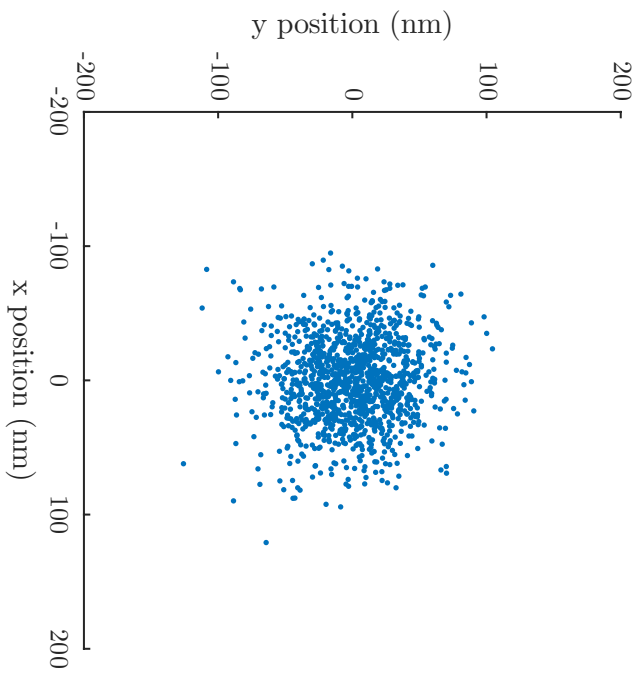

Supplement: S8 Fig — (PDF) [file pone.0172943.s009.pdf]

R estimation evolution

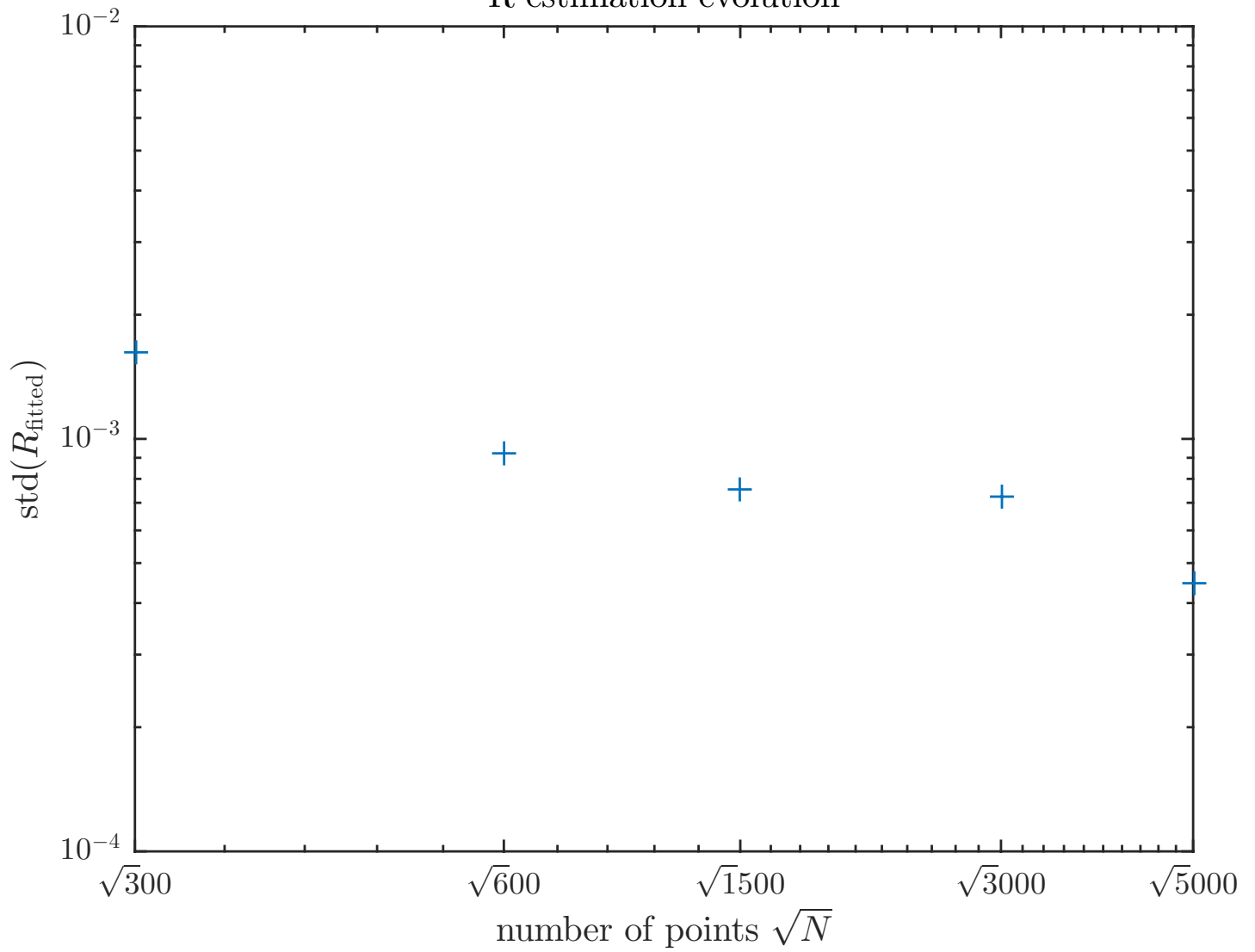

Supplement: S9 Fig — Each cross gives the dispersion of the estimated radii of a cohort of complete spheres around the actual radius value for various density of sampled positions. (PDF) [file pone.0172943.s010.pdf]

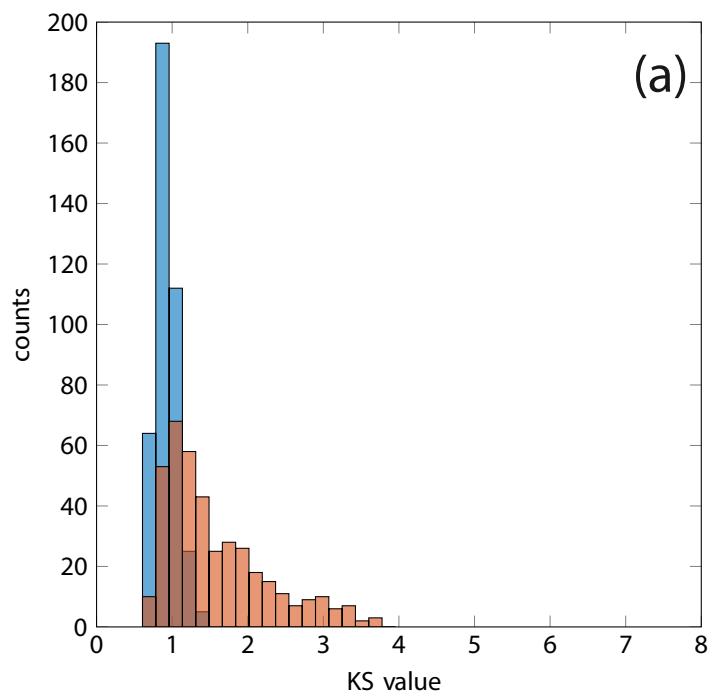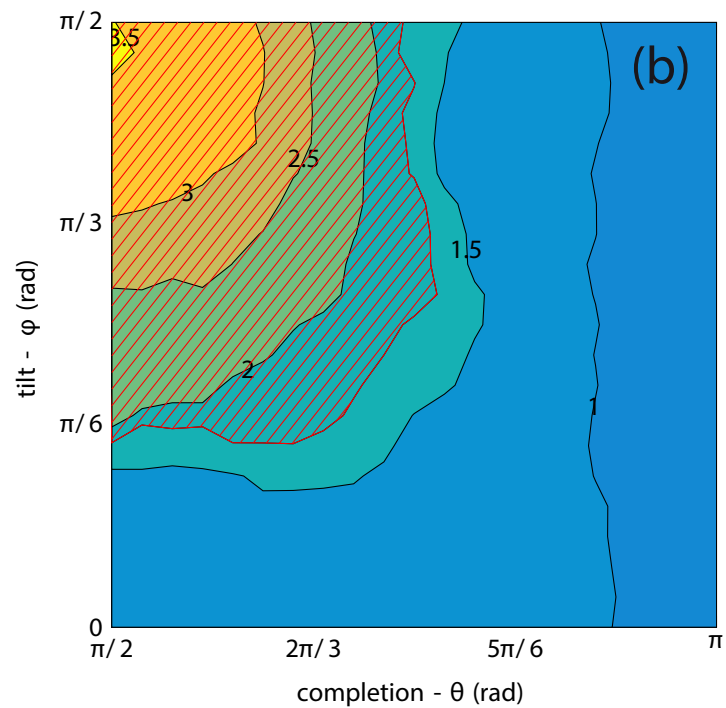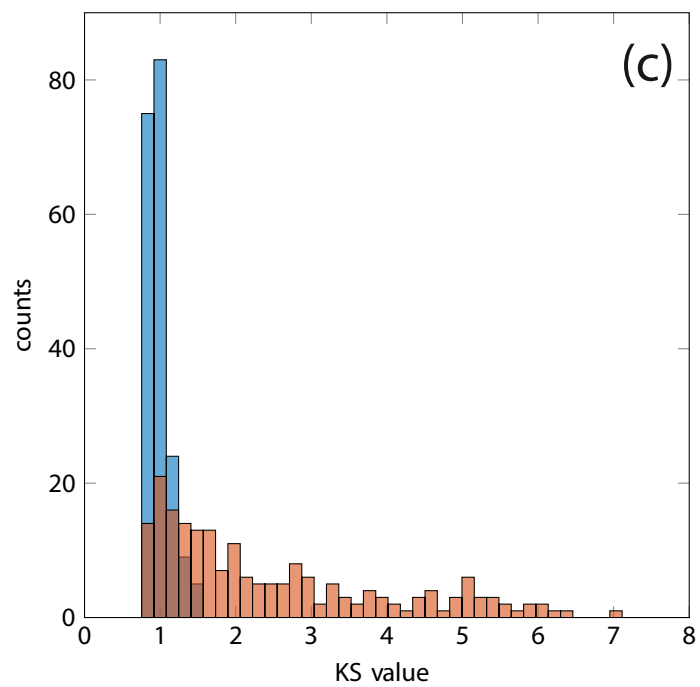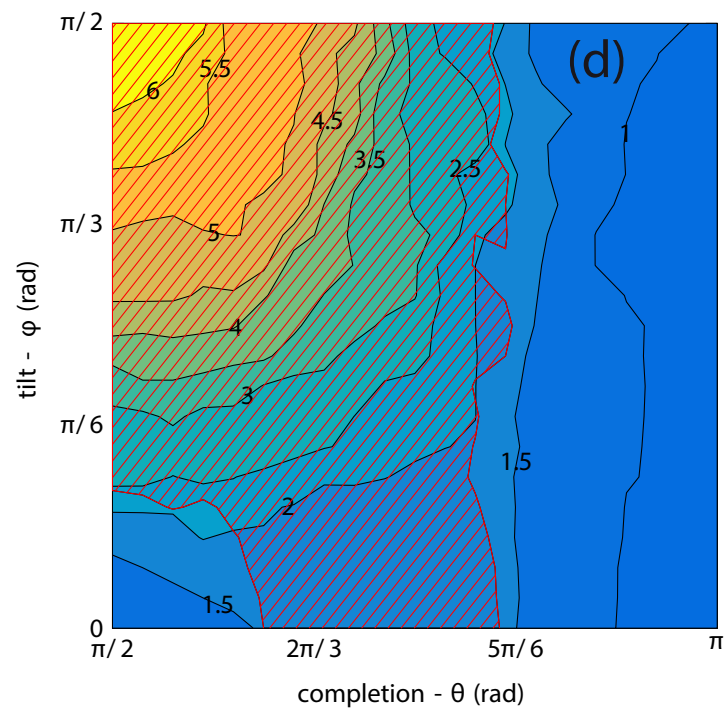

Supplement: S10 Fig — (left) Distribution of normalized KS=Nmax|CDFmodel-CDFempirical| values for complete sphere reconstruction of complete spheres (blue), and truncated spheres. (red). (right) KS distribution mean value (colors and isocontours) for complete sphere reconstruction of simulated truncated spheres as function of initial completion θ and orientation ϕ. The red dashed area shows the region for which 90% of the estimated structure are rejected by the the Kolmogorov-Smirnov test for a normalized KS value of 1.3. (PDF) [file pone.0172943.s011.pdf]

A

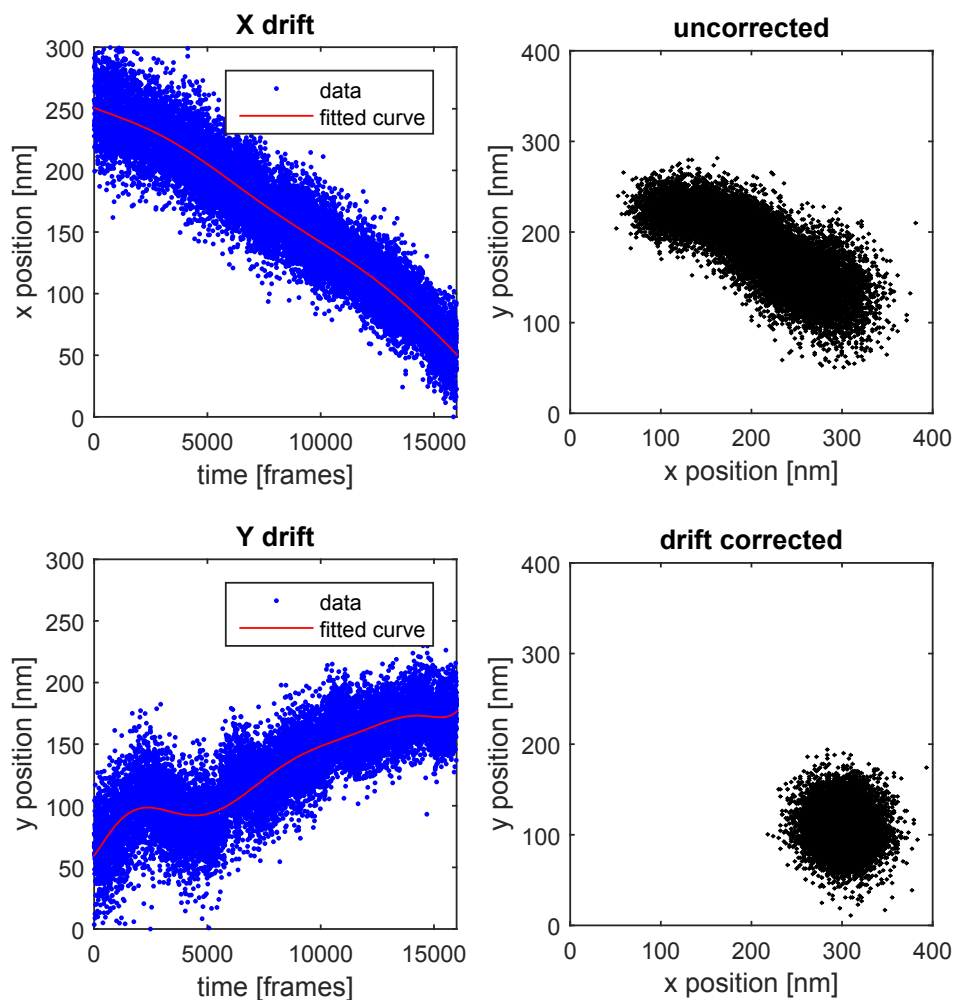

B

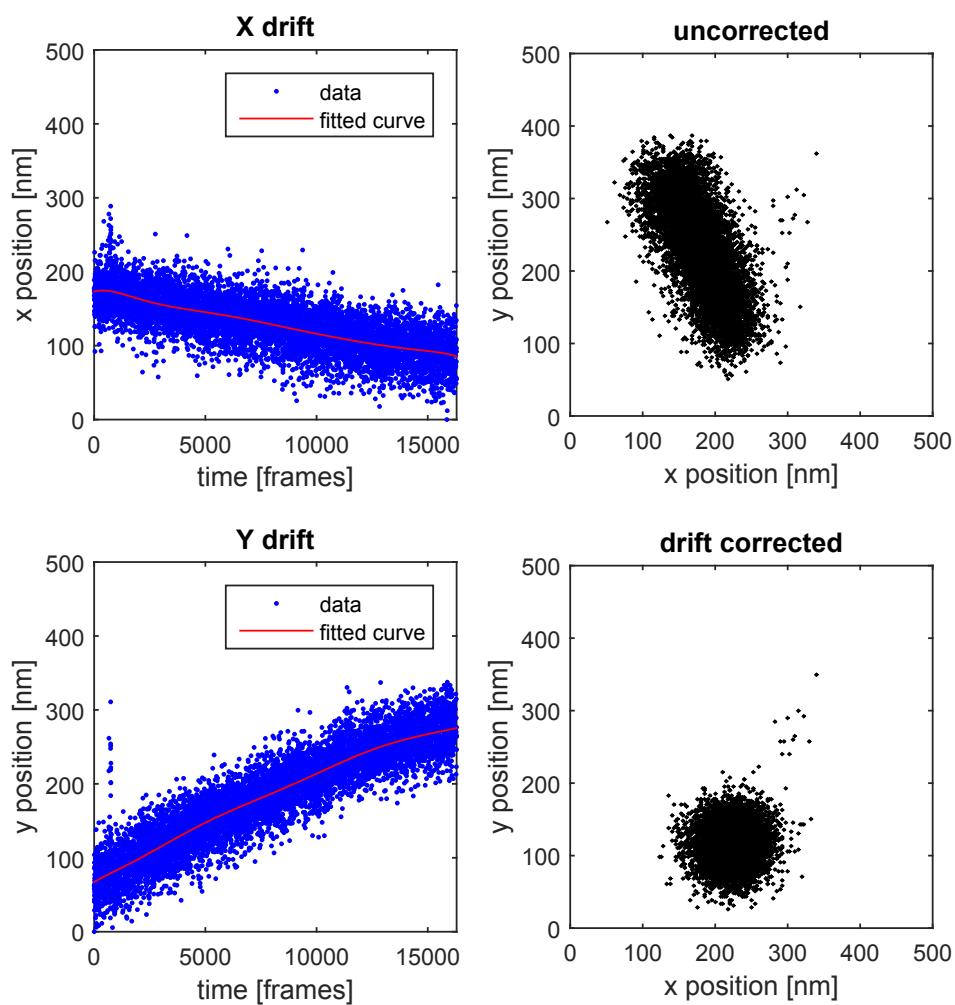

Supplement: S11 Fig — Due their stability, Au fiducials can easily be separated from VLPs within the sample. Shown are two examples from independent experiments (A, B). (1) Individual fiducials were selected (uncorrected), (2) their X and Y position tracked over the full length of the movie (left column) and (3) fitted with a polynomial function (red line, left column). The fitting function is then used to (4) correct all localizations within the sample including the Au fiducial (drift corrected). The accuracy of the fitting (RMSE) was for A: x = 16.7nm, y = 18.4nm and for B: x = 25.1nm, y = 26.6nm. The accuracy corresponds to the lateral localization spread of the drift corrected particle and includes the localization precision. (PDF) [file pone.0172943.s012.pdf]

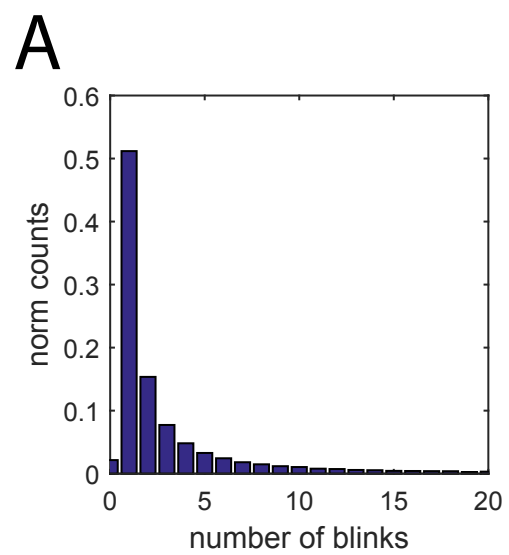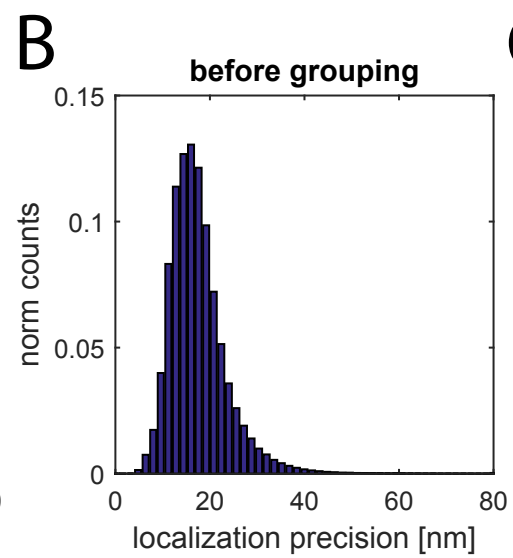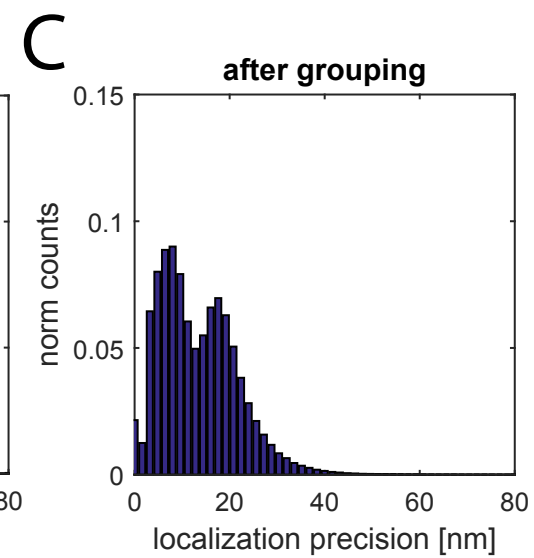

Supplement: S12 Fig — Due to the low number of switching cycles (blinks, on average 3.5) of mEos2 in our experiments (A), an experimental determination of the localization precision is not accurate. Hence, we rely on the theoretical calculation as presented in (Thomson et al., Biophysical Journal, 2002 May 31;82(5):2775–83). We find an average localization precision of σ = 17.6nm for single localizations (B) and σ = 13.4nm for grouped localizations (B). Shown data from three independent experiments. (PDF) [file pone.0172943.s013.pdf]
